# Supplementary material for: Sensitivity analyses for effect modifiers not observed in the target population when generalizing treatment effects from a randomized controlled trial: Assumptions, models, effect scales, data scenarios, and implementation details
Source: PLoS One. 2018 Dec 11;13(12):e0208795. doi: 10.1371/journal.pone.0208795 (PMC6289424; doi:10.1371/journal.pone.0208795)

S1 APPENDIX. SIMULATION RESULTS COMPARING METHOD 1 AND METHOD 2 WHEN THE OUTCOME MODEL IS CORRECTLY OR INCORRECTLY SPECIFIED

(for the paper *Sensitivity analyses for effect modifiers not observed in the target population when generalizing treatment effects from a randomized controlled trial: Assumptions, models, effect scales, data scenarios, and implementation details*)

The simulations here use the same setup as in [1]. In fact, results regarding bias have been reported in [1]. In the current simulations, we track not only bias, but also variance, RMSE, model estimated variance, and confidence interval coverage proportion.

Data generation. All the scenarios include a X variable, a Z variable and a V variable. X is standard normal. Z and V are first generated as multivariate normal with correlations ranging from 0 to ± 0.5, then each is either kept in continuous form or dichotomized. When either Z or V is binary, its prevalence is 0.25 in the trial sample and 0.5 in the target population. When either Z or V is continuous, it has mean 0 in the trial sample and 0.5 in the target population, and variance 1 in both. In the trial, A is randomly assigned to 0 and 1 with equal probability. With regards to the outcome, for the continuous Z and V combination, we use a base model with Z and V as effect modifiers, plus three other models, each with one additional effect modifier from among Z^2, V^2 or ZV:

$$\begin{array}{ll} A. & Y = X + A + Z + V + ZA + VA + \epsilon_Y, \\ B. & Y = X + A + Z + V + ZA + VA + Z^2A + \epsilon_Y, \\ C. & Y = X + A + Z + V + ZA + VA + V^2A + \epsilon_Y, \\ D. & Y = X + A + Z + V + ZA + VA + ZVA + \epsilon_Y, \end{array} \quad \epsilon_Y \sim \mathrm{N}(0, 4).$$

For the continuous Z and binary V combination, we use models A, B and D. For the binary Z and continuous V combination, we use A, C and D. For the binary Z and V combination, we use A and D. For each scenario (combining Z and V types and outcome model), we generate 100,000 pairs of datasets including an $n = 400$ trial sample and an $n = 5000$ target population sample.

Outcome model specification in method implementation. For both methods 1 and 2, in all scenarios we implement the method with the correct outcome model. For scenarios including Z^2, V^2 or ZV as effect modifiers, we also implement the methods with the misspecified outcome model that leaves out these terms and retains only Z and V as effect modifiers; this misspecified model is perhaps the most commonly encountered in practice.

For method 2, the weighting is with respect to X, Z using weights based on a logistic regression of sample membership. Continuous predictors are included using natural splines.

Results. The findings from these simulations are already summarized in the text of the paper. Here we include all the plots of the results, starting on the next page.

References

[1] Trang Quynh Nguyen, Cyrus Ebnesajjad, Stephen R. Cole, and Elizabeth A. Stuart. Sensitivity analysis for an unobserved moderator in RCT-to-target-population generalization of treatment effects. *Annals of Applied Statistics*, 11(1):225–247, 2017.

Figure 1: Bias

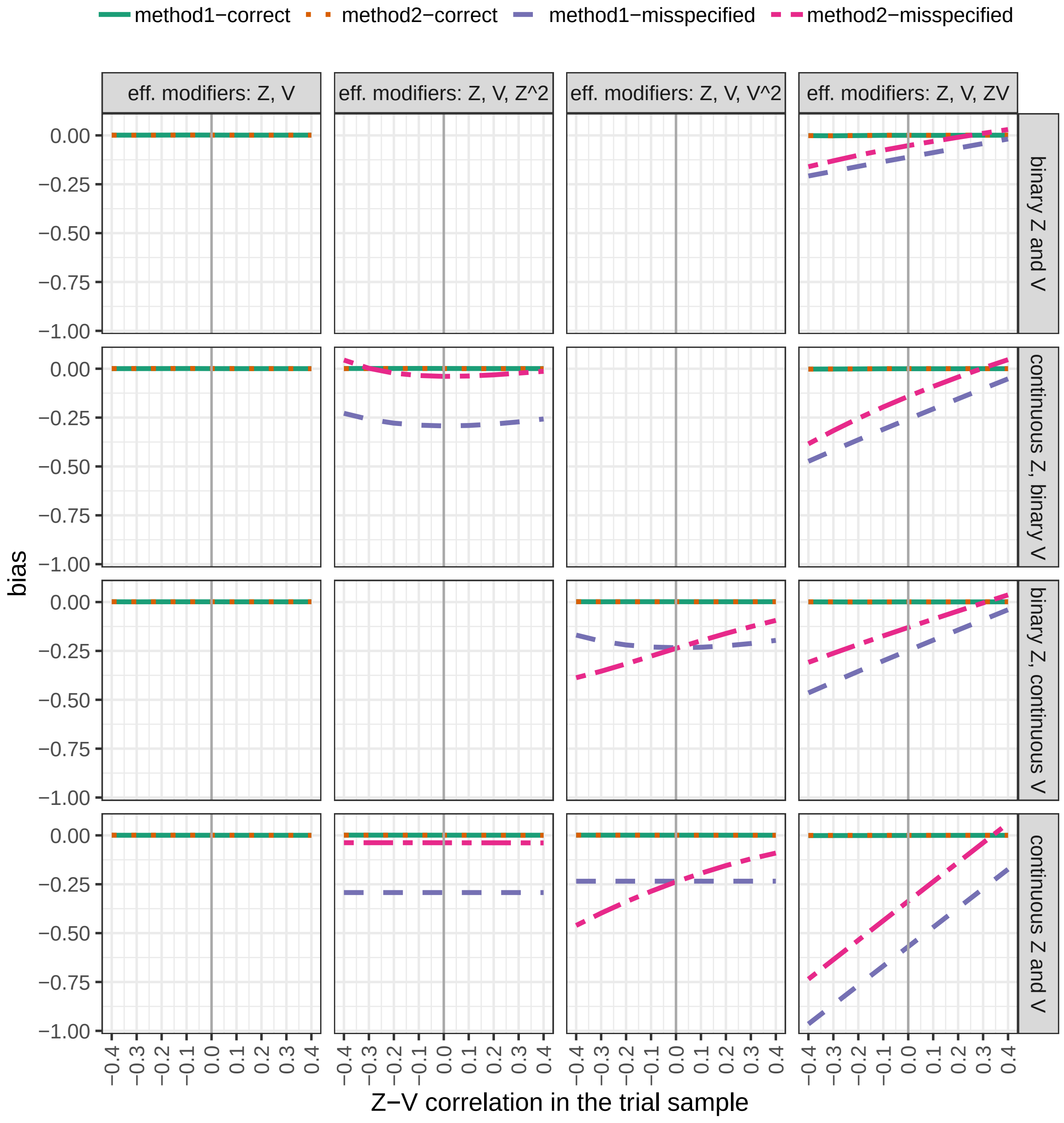

Figure 2: RMSE

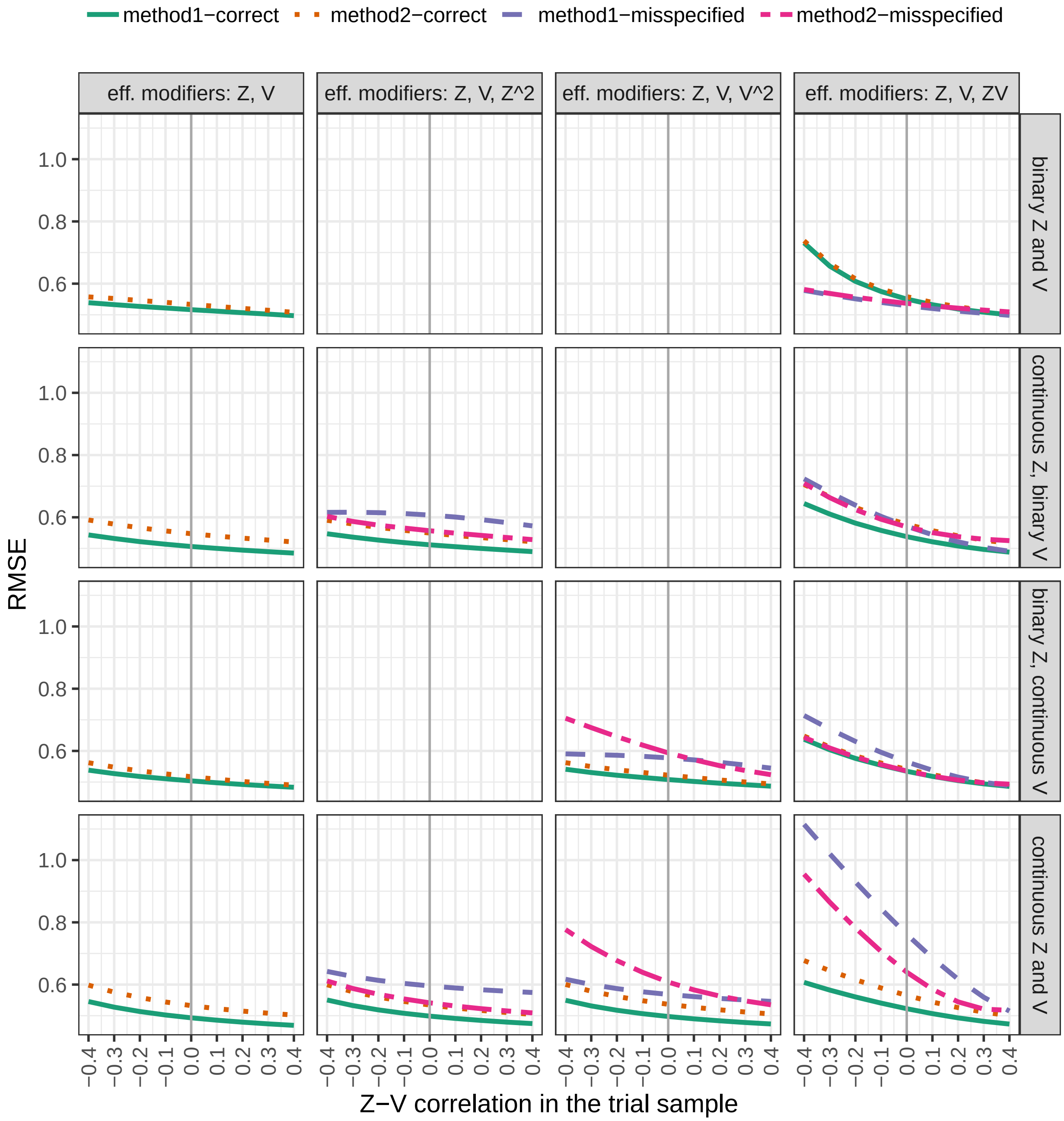

Figure 3: Standard Error

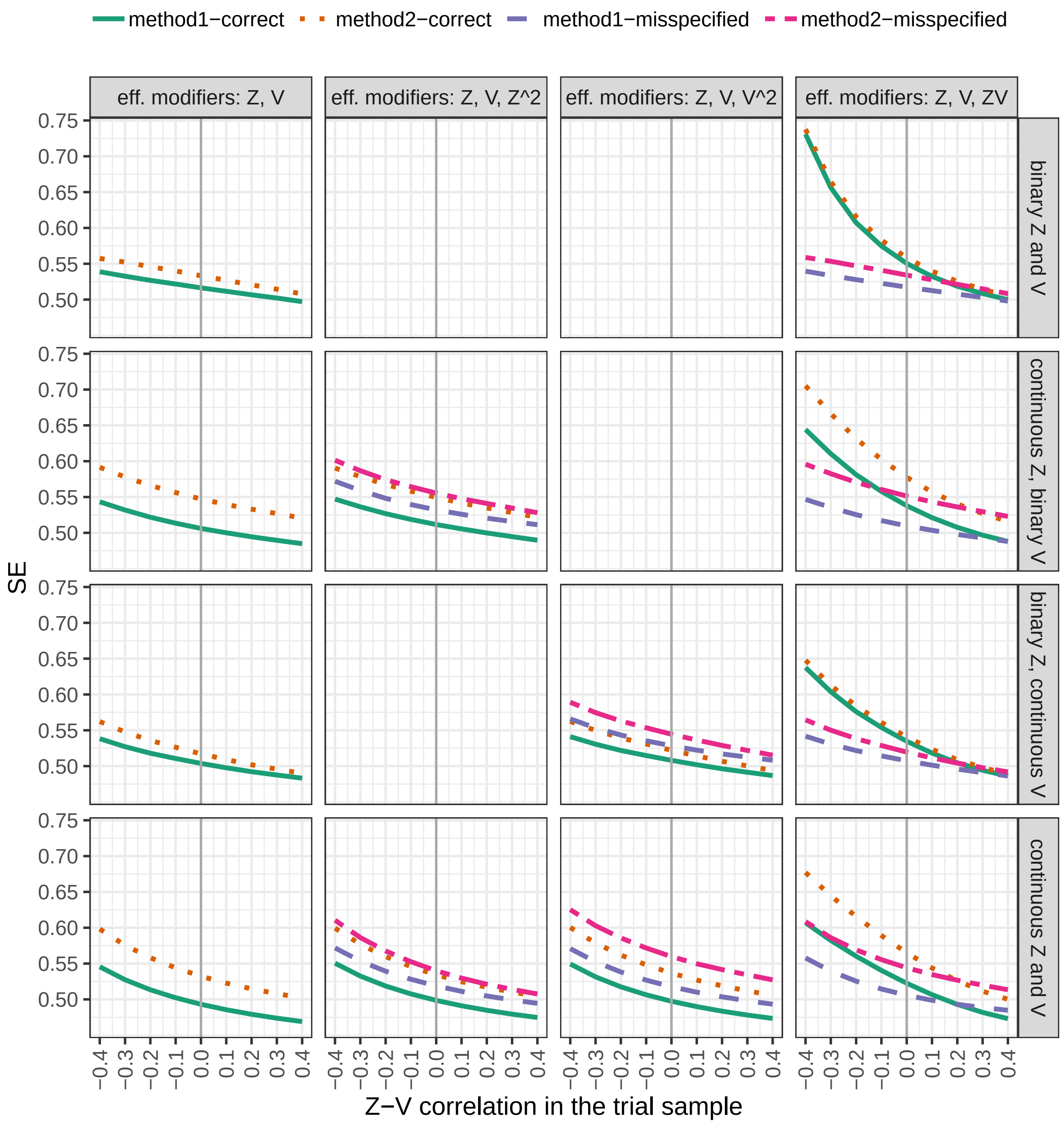

Figure 4: Model-estimated standardeError

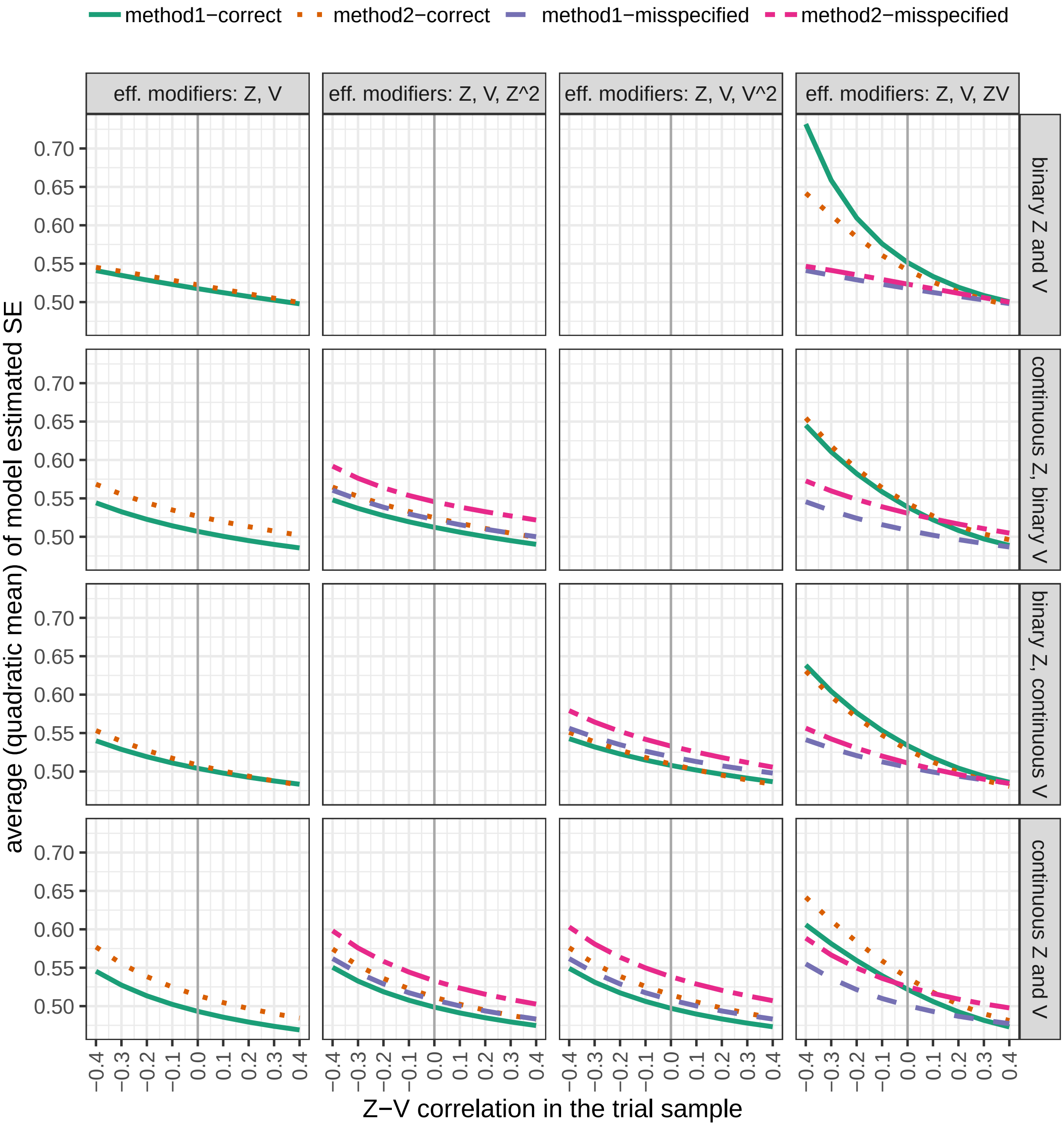

Figure 5: Coverage proportion of 95% confidence interval

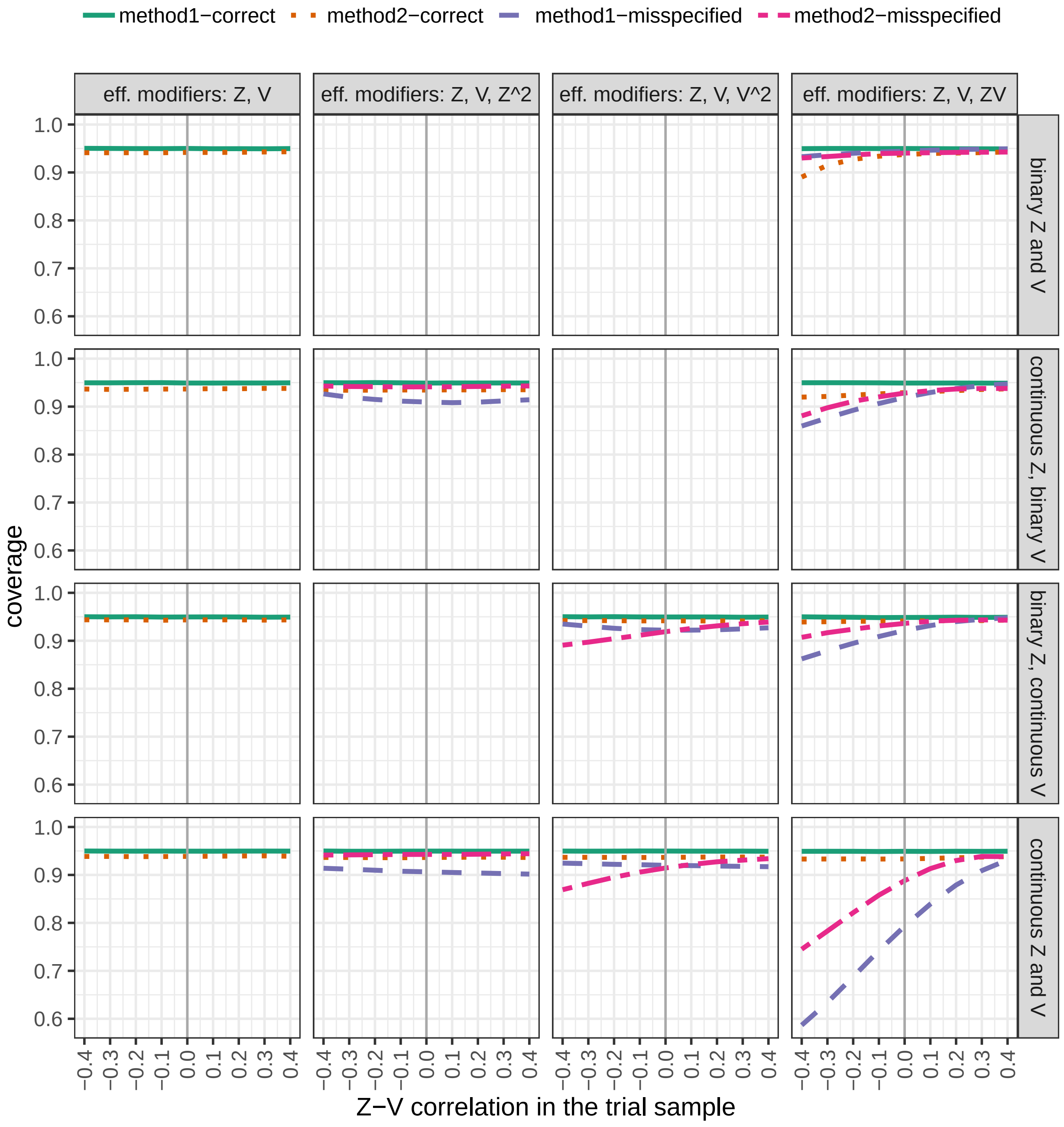

Supplement: S1 Appendix — (PDF) [file pone.0208795.s001.pdf]
