# Supplementary material for: Sensitivity analyses for effect modifiers not observed in the target population when generalizing treatment effects from a randomized controlled trial: Assumptions, models, effect scales, data scenarios, and implementation details
Source: PLoS One. 2018 Dec 11;13(12):e0208795. doi: 10.1371/journal.pone.0208795 (PMC6289424; doi:10.1371/journal.pone.0208795)
Supplement: S2 Appendix — (PDF) [file pone.0208795.s002.pdf]

S2 APPENDIX. A UNIFIED EXPLANATION OF WEIGHTING PROCEDURES FOR THE DIFFERENT DATA SCENARIOS

(for the paper *Sensitivity analyses for effect modifiers not observed in the target population when generalizing treatment effects from a randomized controlled trial: Assumptions, models, effect scales, data scenarios, and implementation details*)

Method 2 presented in this paper call for weighting the trial sample so that it resembles the target population with respect to the distribution of the observed baseline covariates X, Z. Depending on the source of target population data – a full population ($P = 1$) dataset or a representative ($S = 2$) sample, and how it relates to the trial sample (see example scenarios in Fig. 1) – the specific weighting procedures vary. All these weighting procedures, however, relate to one idea we call "ratio-of-probability weighting". We borrow this term from [1], who used it in a different context (mediation analysis), but the term is appropriate for our current purpose. The idea is simple: to weight a sample E so that it resembles sample/population F with respect to the distribution of variables C, we use weights that are ratios of sample membership probabilities conditional on C, $W_i = \frac{\text{P}(\text{E}|C = C_i)}{\text{P}(\text{F}|C = C_i)}$. We now elaborate how this plays out in several data scenarios.

In scenario 1(b), the weights for the $S = 1$ sample to make it resemble the $S = 2$ sample with respect to baseline covariates X, Z are

$$W_i = \frac{\text{P}(S = 2|X = X_i, Z = Z_i)}{\text{P}(S = 1|X = X_i, Z = Z_i)}. \qquad \text{(w1)}$$

With data only from these two samples (assumed to be disjoint), we estimate these weights by stacking the two datasets and fitting a model for sample membership S with X, Z as predictors (e.g., using logistic or another model deemed appropriate), and obtaining for each trial participant a weight that is the model-predicted odds of $S = 2$ vs. $S = 1$ given their X, Z values. Formally, this is an estimate of $\frac{\text{P}\left(S = 2\middle|X = X_i, Z = Z_i, (S = 1 \text{ or } S = 2)\right)}{\text{P}\left(S = 1\middle|X = X_i, Z = Z_i, (S = 1 \text{ or } S = 2)\right)}$, which is equivalent to (w1). This weighting-by-the-odds method [2,3] is analogous to the propensity score weighting version for estimating the average treatment effect on the treated, where control units are weighted by their predicted odds of being in the treatment vs. control condition [4].

In scenario 1(a), the weights for the $S = 1$ sample to make it resemble the $P = 1$ dataset with respect to baseline covariates X, Z are

$$W_i = \frac{\text{P}(P = 1|X = X_i, Z = Z_i)}{\text{P}(S = 1|X = X_i, Z = Z_i)}. \qquad \text{(w2)}$$

If we know which units in the target population dataset are the specific units in the trial, we can fit to the target population dataset a model for trial participation with X, Z as predictors, and use inverse-trial-participation-probability weighting to weight the trial sample up to the population. The weights are estimates of $\frac{1}{\text{P}(S = 1|X = X_i, Z = Z_i, P = 1)} = \frac{\text{P}(P = 1|X = X_i, Z = Z_i, P = 1)}{\text{P}(S = 1|X = X_i, Z = Z_i, P = 1)}$, which are equivalent to (w2). This weighting is analogous to inverse-probability-of-selection weighting in complex survey design [5]. If, on the other hand, the trial participants cannot be linked to their records in the population dataset, we can still estimate these weights by treating

the population dataset as an $S = 2$ dataset, stacking it with the trial dataset and using weighting-by-the-odds as in scenario 1(b). In this case, these artificial "odds" are estimates of $\frac{\text{P}(P=1|X=X_i,Z=Z_i)/[\text{P}(P=1|X=X_i,Z=Z_i)+\text{P}(S=1|X=X_i,Z=Z_i)]}{\text{P}(S=1|X=X_i,Z=Z_i)/[\text{P}(P=1|X=X_i,Z=Z_i)+\text{P}(S=1|X=X_i,Z=Z_i)]}$, which are equivalent to (w2).

In a slightly different scenario where the trial sample has been drawn from within an observational sample that represents the target population, we proceed as in scenario 1(a), but treating the observational sample as if it were the population.

When the problem is one of *transportation*, where the trial sample is not part of the target population, the same weight formula (w2) or (w1) applies. Given a $P = 1$ or $S = 2$ dataset for the target population, we need to stack it with the $S = 1$ dataset, and use weighting-by-the-odds.

In the description of the sensitivity analyses, we mentioned that weighting-based sensitivity analyses are used only if a target population dataset (either $P = 1$ or $S = 2$) is available. To be precise, in a special case where there is no target population dataset, but information is available on the target population distribution of $\{X, Z\}$ (e.g., from a census or a prior population estimation exercise that reported these variables' joint distribution), weighting may also be implemented, using $W_i = \frac{\text{P}(X=X_i,Z=Z_i|P=1)}{\text{P}(X=X_i,Z=Z_i|S=1)}$, which are proportional to (w2). Here the numerator and denominator are the prevalences/densities of the $\{X_i, Z_i\}$ pattern in the target population and in the trial sample, respectively. This is only recommended for discrete $\{X, Z\}$ with a small number of combined categories, because beyond this situation, it is generally hard to estimate the denominator and the available estimates for the numerator may not be reliable.

To sum up, in most data scenarios where a dataset for/representing the target population is available, weighting the trial sample to make it resemble the target population involves data stacking and weighting-by-the-odds. The exception is when the trial sample is part of and can be identified within the population dataset, in which case inverse-probability weighting is used. If only summary statistics are available for the target population, the sensitivity analyses that involve weighting will generally not be used, except the very special case mentioned in the previous paragraph.

References:

1 Hong G. Ratio of mediator probability weighting for estimating natural direct and indirect effects. *Proc Am Stat Assoc Biometrics Sect* 2010;:2401–15.

2 Kern HL, Stuart EA, Hill JL, *et al.* Assessing methods for generalizing experimental impact estimates to target samples. *J Res Educ Eff* 2016;**9**:103–27. doi:10.1080/19345747.2015.1060282

3 Westreich D, Edwards JK, Lesko CR, *et al.* Transportability of trial results using inverse odds of sampling weights. *Am J Epidemiol*

4 Hirano K, Imbens GW, Ridder G. Efficient estimation of average treatment effects using the estimated propensity score. *Econometrica* 2003;**71**:1161–89. doi:10.1111/1468-0262.00442

5 Horvitz D, Thompson D. A generalization of sampling without replacement from a finite universe. *J Am Stat Assoc* 1952;**47**:663–85.
