# Supplementary material for: Sensitivity analyses for effect modifiers not observed in the target population when generalizing treatment effects from a randomized controlled trial: Assumptions, models, effect scales, data scenarios, and implementation details
Source: PLoS One. 2018 Dec 11;13(12):e0208795. doi: 10.1371/journal.pone.0208795 (PMC6289424; doi:10.1371/journal.pone.0208795)
Supplement: S3 Appendix — (PDF) [file pone.0208795.s003.pdf]

S3 APPENDIX. ADDITIONAL MATERIAL ON EFFECT MODIFIERS NOT OBSERVED IN THE TRIAL

(for the paper *Sensitivity analyses for effect modifiers not observed in the target population when generalizing treatment effects from a randomized controlled trial: Assumptions, models, effect scales, data scenarios, and implementation details*)

Denote the effect modifier not observed in the trial by U. As stated in the text, U can be a specific variable (e.g., addiction severity) or a generic representation of unknown factors. Mimicking the V case, assume the causal model

$$\text{E}[Y_i(a)] = \beta_o + \beta_a a + \beta_x X_i + \beta_z Z_i + \beta_{za} Z_i a + \beta_u U_i + \beta_{ua} U_i a.$$

The TATE formula copied from the V case (replacing V with U),

$$\text{TATE} = \beta_a + \beta_{za}\text{E}[Z|P=1] + \beta_{ua}\text{E}[U|P=1],$$

is not helpful, as it requires estimates for $\beta_a, \beta_{za}, \beta_{ua}$, all of which are not identified from trial data because we do not observe U. Let's try to see if we can do something else that might work better. The following (essentially a bias formula) is obtained by comparing the formulas for TATE and SATE.

$$\text{TATE} = \text{SATE} + \beta_{za}\underbrace{\{\text{E}[Z|P=1] - \text{E}[Z|S=1]\}}_{\Delta_Z} + \beta_{ua}\underbrace{\{\text{E}[U|P=1] - \text{E}[U|S=1]\}}_{\Delta_U}.$$

Here SATE is identified from trial data. So is Δ_Z as Z is observed in both the trial and the target population. Suppose we are willing to treat β_{ua} (effect modification by U) and Δ_U (the difference in mean U between the target population and the trial) as sensitivity parameters for which we will specify ranges, as these two parameters are meaningful and somewhat imaginable. We are still stuck with an unidentified parameter, β_{za}.

If we use the weighting approach, and manage to equate mean Z between the trial and the target population, the second term in the formula vanishes, so we no longer have to deal with β_{za}. However, the other terms also change. Instead of SATE, we now can estimate a weighted ATE in the weighted trial sample, which is fine. The sensitivity parameter β_{ua} retains its meaning, so it does not pose a problem. However, in the place of $\text{E}[U|S=1]$, we now have the weighted trial mean U, so instead of the Δ_U above, we now have the difference in mean U between the target population and the weighted trial sample, an obscure quantity that is not as imaginable and meaningful as Δ_U, so it is not suitable to serve as a sensitivity parameter.

The conclusion then is that the sensitivity analyses developed for the V case do not extend to the U case!

Correction of previously published results: Our previous paper [1] claimed that the methods do extend to the U case if we consider a special U that is the *remaining composite effect modifier after accounting for Z*, i.e., it captures all effect modification forces other than Z and it is independent of X, Z (intuitively it is a combination of all the remaining effect modifiers and X, Z have been "regressed out" of it), then due to this independence, a regression model without U fit to the trial sample can recover β_{za}, so the TATE formula above can be used. Also due to this independence, weighting based on X, Z does not change the distribution of U, so after weighting, we still have the simple Δ_U in the TATE formula, without having to deal with a weighted trial sample mean U that is different from the original trial sample mean U. This reasoning is flawed. Both parts of this reasoning hangs on the idea of a composite U independent of X, Z. The problem is with Z and U both differentially distributed between the trial sample and the target population (the motivating factor for sensitivity analysis for U), the association of Z and U is generally different between the trial sample and the target population due to collider bias when conditioning on sample membership. Thus independence of U and Z does not exists in both places. It is independence in the trial sample that would give the result of recovering β_{za} and weighting not changing the distribution of U, but it needs to be independence in the target population to make the notion of U meaningful as we are interested in the universe

that is the target population, not just one specific piece of it that is the trial sample. In addition, there is another flaw, that regressing X, Z out of U results in U being uncorrelated with X, Z, not independence. If we replace independence with uncorrelatedness, then we also lose the claim that weighting based on X, Z does not change the distribution of U.

References

[1] Trang Quynh Nguyen, Cyrus Ebnesajjad, Stephen R. Cole, and Elizabeth A. Stuart. Sensitivity analysis for an unobserved moderator in RCT-to-target-population generalization of treatment effects. *Annals of Applied Statistics*, 11(1):225–247, 2017.
