# Supplementary material for: Sensitivity analyses for effect modifiers not observed in the target population when generalizing treatment effects from a randomized controlled trial: Assumptions, models, effect scales, data scenarios, and implementation details
Source: PLoS One. 2018 Dec 11;13(12):e0208795. doi: 10.1371/journal.pone.0208795 (PMC6289424; doi:10.1371/journal.pone.0208795)
Supplement: S4 Appendix — (PDF) [file pone.0208795.s004.pdf]

S4 APPENDIX. THE SENSITIVITY ANALYSES WHEN RANDOM INTERCEPTS MODELS ARE USED

(for the paper *Sensitivity analyses for effect modifiers not observed in the target population when generalizing treatment effects from a randomized controlled trial: Assumptions, models, effect scales, data scenarios, and implementation details*)

When the trial data are analyzed using random intercepts models, it is natural to define treatment effects as differences in pre-to-post change in outcome between treatment and control (or effect on 'potential outcome change'). It turns out that this is equivalent to effect on potential outcome post-treatment. Take any individual i. Let Y_{i1} denote individual i's pre-treatment outcome measure, and $Y_{i2}(a)$ denote individual i's potential outcome post-treatment if treatment is set to a. Denote potential outcome change under treatment a as $H_i(a)$. Then $H_i(a) = Y_{i2}(a) - Y_{i1}$. The individual treatment effect defined as difference in potential outcome change is formally $\text{TE}_i = \text{E}[H_i(1) - H_i(0)]$, which is equal to $\text{E}[Y_{i2}(1) - Y_{i2}(0)]$.

In this case, the same sensitivity analyses apply, with minor modifications to the models fit and the estimates used. The explanation below references the simpler case where treatment effect is the difference in potential outcomes post-treatment that we used to describe the methods in the main text of the paper; for brevity, we will refer to that case as "the simple case".

To estimate the ATE from a trial with both baseline and post-treatment measures of the outcome, the simplest model that can be used is the model without covariates,

$$\text{E}\left[Y_{ij}|F_{ij}, A_i\right] = c_{0i} + \gamma_0 + \gamma_a A_i + \gamma_f F_{ij} + \gamma_{fa} F_{ij} A_i$$

where i indexes the person, j indexes the observation (each person has two observations), T indicates treatment condition assigned, F indicates that the outcome is post- versus pre-treatment, and c_{0i} is the departure of individual i's intercept from the mean intercept γ_0. In this model, γ_f and $\left(\gamma_f + \gamma_{fa}\right)$ respectively estimate the average pre-to-post change in outcome in the control group and in the treatment group; γ_{fa} estimates the difference between these two average changes, i.e., SATE. This estimator is analogous to estimating SATE using the difference in mean outcome between the two conditions in the simple case.

Note that in this model the stand-alone treatment term A is included just to allow the pre-treatment outcome to differ between the two treatment conditions. Its coefficient (γ_a) is usually small because due to randomization, its expectation is zero.

Another way to estimate SATE is fit a model that adjusts for baseline covariates but not letting the covariates interact with treatment; this is analogous to the regression of the outcome on treatment and covariates in the simple case. With X and Z, the model is

$$\text{E}\left[Y_{ij}|F_{ij}, A_i, X_i, Z_i\right] = c_{0i} + (\gamma_0 + \gamma_x X_i + \gamma_z Z_i) + \gamma_a A_i + \left(\gamma_f + \gamma_{xf} X_i + \gamma_{zf} Z_i\right) F_{ij} + \gamma_{fa} F_{ij} A_i.$$

Note that this model includes the possibility that some baseline covariates may influence change in outcome that is not due to treatment, via the interaction terms of X and Z with F; this does not

mean they modify treatment effect. Treatment effect, again, is represented by γ_{pa} and is not allowed to vary as a function of baseline covariates, i.e., it is SATE. The model can be written in a more conventional form,

$$\mathrm{E}\left[Y_{ij}|F_{ij}, A_i, X_i, Z_i\right] = c_{0i} + \gamma_0 + \gamma_a A_i + \gamma_f F_{ij} + \gamma_{fa} F_{ij} A_i + \gamma_x X_i + \gamma_z Z_i + \gamma_{xf} X_i F_{ij} + \gamma_{zf} Z_i F_{ij}.$$

The calibration of TATE and the sensitivity analyses rely on a model that captures treatment effect heterogeneity. With X and Z, the potential outcomes model is

$$\mathrm{E}\left[Y_{ij}(a)\right] = b_{0i} + (\beta_0 + \beta_x X_i + \beta_z Z_i) + \beta_a A + \left(\beta_f + \beta_{xf} X_i + \beta_{zf} Z_i\right) F_{ij} + \left(\beta_{fa} + \beta_{zfa} Z_i\right) F_{ij} a,$$

in which treatment effect modification by Z is represented by β_{zfa}. Written in a more conventional form,

$$\mathrm{E}\left[Y_{ij}(a)\right] = b_{0i} + \beta_0 + \beta_a a + \beta_f F_{ij} + \beta_{fa} F_i a + \beta_x X_i + \beta_z Z_i + \beta_{xf} X_i F_{ij} + \beta_{zf} Z_i F_{ij} + \beta_{zfa} Z_i F_{ij} a.$$

The individual treatment effect has expectation $\beta_{fa} + \beta_{zfa} Z_i$, and $\mathrm{TATE} = \beta_{fa} + \beta_{zfa} \mathrm{E}[Z|P = 1]$. The same sensitivity analyses as in the simple case apply, with the following changes in the regression model and the TATE formula.

With effect modifier V observed in the trial but not the target population (and effect modifier Z observed in both samples), the effect modification regression model is

$$\begin{aligned}\mathrm{E}\left[Y_{ij}|F_{ij}, A_i, X_i, Z_i, V_i\right] &= b_{0i} + \beta_0 + \beta_a A_i + \beta_f F_{ij} + \beta_{fa} F_{ij} A_i + \beta_x X_i + \beta_z Z_i + \beta_v V_i + \beta_{xf} X_i F_{ij} + \beta_{zf} Z_i F_{ij} \\ &\quad + \beta_{vf} V_i F_{ij} + \beta_{zfa} Z_i F_{ij} A_i + \beta_{vfa} V_i F_{ij} A_i\end{aligned}$$

(interaction terms of X, Z, V variables with F may be removed if their coefficients are zero). The formula for TATE is

$$\mathrm{TATE} = \beta_{fa} + \beta_{zfa} \mathrm{E}[Z|P = 1] + \beta_{vfa} \mathrm{E}[V|P = 1].$$
