# Supplementary material for: Sensitivity analyses for effect modifiers not observed in the target population when generalizing treatment effects from a randomized controlled trial: Assumptions, models, effect scales, data scenarios, and implementation details
Source: PLoS One. 2018 Dec 11;13(12):e0208795. doi: 10.1371/journal.pone.0208795 (PMC6289424; doi:10.1371/journal.pone.0208795)
Supplement: S5 Appendix — (PDF) [file pone.0208795.s005.pdf]

S5 APPENDIX. ADDITIONAL DETAILS ON EXTENSION 2 FOR MULTIPLICATIVE EFFECTS AND LOG/LOGIT LINK MODELS

(for the paper *Sensitivity analyses for effect modifiers not observed in the target population when generalizing treatment effects from a randomized controlled trial: Assumptions, models, effect scales, data scenarios, and implementation details*)

The main text of the paper presents the case of a binary outcome and an assumed logit causal model. The reasoning for other cases with log/logit link models and multiplicative effects is similar. While it is somewhat repetitive, for clarity, we list the models and effects for a couple of other cases.

Binary outcome, log-probability model, risk ratio scale effects

The causal model:

$$\log\{\Pr[Y_i(a)=1]\} = \beta_0 + \beta_a a + \beta_x X_i + \beta_z Z_i + \beta_{za} Z_i a + \beta_v V_i + \beta_{va} V_i a.$$

The regression model:

$$\log\{\Pr[Y=1|A,X,Z,V]\} = \beta_0 + \beta_a a + \beta_x X + \beta_z Z + \beta_{za} Z_i A + \beta_v V + \beta_{va} VA.$$

Individual effect on the risk ratio (RR) and log RR scales:

$$\begin{aligned} \text{TE}_i^{\text{RR}} &:= \frac{\Pr[Y_i(1)=1]}{\Pr[Y_i(0)=1]} = \exp(\beta_a + \beta_{za} Z_i + \beta_{va} V_i), \\ \text{TE}_i^{\text{log\_RR}} &:= \log(\text{TE}_i^{\text{RR}}) = \beta_a + \beta_{za} Z_i + \beta_{va} V_i. \end{aligned}$$

TATE as arithmetic mean of individual effects on the log RR scale and geometric mean of individual effects on the RR scale:

$$\begin{aligned} \text{TATE}^{\text{log\_RR}} &= \beta_a + \beta_{za}\text{E}[Z|P=1] + \beta_{va}\text{E}[V|P=1], \\ \text{TATE}^{\text{RR}} &= \exp(\beta_a + \beta_{za}\text{E}[Z|P=1] + \beta_{va}\text{E}[V|P=1]). \end{aligned}$$

Count outcome, log link model, mean/rate ratio scale effects

The causal model:

$$\log\{\text{E}[Y_i(a)]\} = \beta_0 + \beta_a a + \beta_x X_i + \beta_z Z_i + \beta_{za} Z_i a + \beta_v V_i + \beta_{va} V_i a.$$

The regression model:

$$\log\{\text{E}[Y|A,X,Z,V]\} = \beta_0 + \beta_a a + \beta_x X + \beta_z Z + \beta_{za} Z_i A + \beta_v V + \beta_{va} VA.$$

Individual effect on the mean ratio (MR) (or rate ratio) and log MR (or log rate ratio) scales:

$$\begin{aligned} \text{TE}_i^{\text{MR}} &:= \frac{\text{E}[Y_i(1)]}{\text{E}[Y_i(0)]} = \exp(\beta_a + \beta_{za} Z_i + \beta_{va} V_i), \\ \text{TE}_i^{\text{log\_MR}} &:= \log(\text{TE}_i^{\text{MR}}) = \beta_a + \beta_{za} Z_i + \beta_{va} V_i. \end{aligned}$$

TATE as arithmetic mean of individual effects on the log MR (or log rate ratio) scale and geometric mean of individual effects on the MR (or rate ratio) scale:

$$\begin{aligned} \text{TATE}^{\text{log\_MR}} &= \beta_a + \beta_{za}\text{E}[Z|P=1] + \beta_{va}\text{E}[V|P=1], \\ \text{TATE}^{\text{MR}} &= \exp(\beta_a + \beta_{za}\text{E}[Z|P=1] + \beta_{va}\text{E}[V|P=1]). \end{aligned}$$

Relating the average causal OR and the conditional OR estimated by logistic regression with main effects only

This discussion about average causal effects is more general than the specific case of the trial sample or target population in this paper. Therefore we drop the reference to the population/sample, and talk about the ATE in a generic way.

Before considering multiplicative effects, let's refer back to the case of additive effects based on a linear model. Clearly, the individual effects vary, as they depend on the individual's Z_i and V_i. We can fit a correct linear regression model (with A, X, Z, ZA, V, VA as predictors, predict the individual treatment effects using $\beta_a + \beta_{za}Z_i + \beta_{va}V_i$ and averaging those to estimate the ATE, which equals $\beta_a + \beta_{za}\text{E}Z + \beta_{va}\text{E}V$. On the other hand, if we fit a linear regression model with A, X, Z, V as predictors (the model with main effects only), then the regression coefficient γ_a of A in this model is equivalent to $\beta_a + \beta_{za}\text{E}Z + \beta_{va}\text{E}V$, which happens to be the ATE. This equivalence is a feature of linear models. Another way to think about this is that the coefficient of A in the model with main effects only estimates the effect of treatment on the outcome with a constraint that the treatment effect is the same for every individual. While for each individual, the estimate is off by some degree, on average, it is right, as it is equal to the average of the true individual effects.

That is, the linear regression model with main effects only is an unbiased estimate of the ATE – a fact that we already know and have used again and again in the paper for the estimation of SATE.

Now let's translate this reasoning to the OR case.

The average causal OR is the average (= geometric mean) of individual ORs which vary as they depend on Z_i, V_i. The logistic regression model with main effects only estimates treatment effects under a constraint (assumption) that treatment effects do not vary across individuals. Since some individuals have higher OR and some have lower, the estimate under this constraint is almost guaranteed to be off for the individuals, but reflects some sort of average over them. Like in the linear model case above, we can think of the OR estimated by this model as an estimate of the average of the individual effects, i.e., the average causal OR. However, it is only an approximate estimate because the model with main effects only has fewer predictors than the correct model with interaction effects, and with logistic regression dropping predictors leads to less variation in the outcome being explained, which tends to deflate the log OR; this is a problem with ORs called non-collapsibility. Therefore, the conditional OR estimated by logistic regression with main effects is in the spirit of estimating the average causal OR, but due to this reduction in variance explained, it tends to underestimate the average causal OR.
