# Supplementary material for: Sensitivity analyses for effect modifiers not observed in the target population when generalizing treatment effects from a randomized controlled trial: Assumptions, models, effect scales, data scenarios, and implementation details
Source: PLoS One. 2018 Dec 11;13(12):e0208795. doi: 10.1371/journal.pone.0208795 (PMC6289424; doi:10.1371/journal.pone.0208795)
Supplement: S6 Appendix — (HTML) [file pone.0208795.s006.html]

Sensitivity Analysis for Unobserved Effect Modifiers in Generalization: Illustration


# Sensitivity Analysis for Unobserved Effect Modifiers in Generalization: Illustration

# 1 READ ME

This document is the main appendix (S6 appendix) covers the illustrative example in the paper

*Sensitivity analyses for effect modifiers not observed in the target population when generalizing treatment effects from a randomized controlled trial: Assumptions, models, effect scales, data scenarios and implementation details*

by Trang Quynh Nguyen, Ben Ackerman, Ian Schmid, Stephen R. Cole, and Elizabeth A. Stuart.

Two data files are used for the illustration: one for the trial (synthetictrial.csv, provided in the S8 appendix), and one for the target population (us.csv, provided in the S9 appendix).

All code for data manipulation and analysis in R are included in the current document. Readers can skip straight to section 4. Synthetic trial data analysis. Sections 2 and 3 are included only for completeness, so interested readers can see how the synthetic trial data were created.

This document mentions the real trial data, only as the basis of the creation of the synthetic data used in the illustrative example. The real data were NOT part of the example and of this paper, and thus are not shared.

In addition to the R code in this document, we attach Stata code in the file StataFits.do (S7 appendix). This is used to obtain correct variance estimates when fitting mixed effects models with probability weights. The R package lme4 used in this document was not built to handle probability weights, therefore it is only good for the point estimates when models are fit with weights that are probability weights.

```
library(tidyverse)
library(lme4)
library(lmerTest)
library(survey)
```

# 2 Real trial data analysis

## 2.1 Data wrangling

Input two data files that are available from the trial: the main dataset without CD4 count (one observation per individual) and the CD4 count dataset (multiple counts, thus multiple observations, per person).

```
main <- read.table(here::here("data", "actg320.27feb15.dat"), header=FALSE,
                   col.names = c("id", "new", "event", "event.time",
                                 "age", "fem", "nwhite", "entry"))

main <- main %>% select(id, entry, new, age, fem, nwhite)

cd4 <- read.table(here::here("data", "actg320cd4.27feb15.dat"),
                  header=FALSE, col.names = c("id", "cd4", "date"))
```

Extract baseline and post-treatment CD4 counts and baseline status of severe immune suppression (sis), and restrict dataset to those with baseline and post-treatment CD4 counts.

```
cd4 <- cd4 %>% inner_join(main %>% select(id, entry), by = "id")

cd4.base <- cd4 %>%
    filter(date >= entry - 10 & date <= entry + 10) %>%
    group_by(id) %>%
    summarize(sis = as.integer(min(cd4) <= 50),
              cd4.base = mean(cd4))

cd4.post <- cd4 %>%
    filter(date >= (entry + 61) - 10 & date <= (entry + 61) + 10) %>%
    group_by(id) %>%
    summarize(cd4.post = mean(cd4))

dat <- main %>%
    inner_join(cd4.base, by = "id") %>%
    inner_join(cd4.post, by = "id") %>%
    select(id, new, cd4.base, cd4.post, age, fem, nwhite, sis)

rm(cd4, cd4.base, cd4.post)

names(dat); dim(dat)
```

```
## [1] "id"       "new"      "cd4.base" "cd4.post" "age"      "fem"      "nwhite"   "sis"
```

```
## [1] 933   8
```

Reshape to long data for multilevel modeling later.

```
long <- dat %>%
    gather("post", "cd4",
           -c(id, new, age, fem, nwhite, sis)) %>%
    mutate(post = as.integer(post == "cd4.post"))

names(long); dim(long)
```

```
## [1] "id"     "new"    "age"    "fem"    "nwhite" "sis"    "post"   "cd4"
```

```
## [1] 1866    8
```

### 2.1.1 Additional functions for later data wrangling

```
cross.dummy.race.sis <- function(data) {
    data %>% 
        mutate(nwhite.sis = interaction(nwhite, sis)) %>%
        fastDummies::dummy_cols(select_columns = "nwhite.sis") %>%
        rename(white.nosis    = "nwhite.sis_0.0",
               white.sis      = "nwhite.sis_0.1",
               nonwhite.nosis = "nwhite.sis_1.0",
               nonwhite.sis   = "nwhite.sis_1.1") %>%
        mutate(nwhite.sis.plot = factor(nwhite.sis,
                                        levels = c("0.0", "1.0", "0.1", "1.1"),
                                        labels = c("white.nosis", 
                                                   "nonwhite.nosis",
                                                   "white.sis",
                                                   "nonwhite.sis")))
}
```

```
construct.newdata <- function(dat) {
    newdat <- data.frame(
        nwhite   = c(0, 0, 1, 1),
        sis      = c(0, 1, 0, 1),
        cd4.base = 
            c(dat %>% filter(nwhite==0, sis==0) %>% pull(cd4.base) %>% mean(),
              dat %>% filter(nwhite==0, sis==1) %>% pull(cd4.base) %>% mean(),
              dat %>% filter(nwhite==1, sis==0) %>% pull(cd4.base) %>% mean(),
              dat %>% filter(nwhite==1, sis==1) %>% pull(cd4.base) %>% mean()),
        age      =
            c(dat %>% filter(nwhite==0, sis==0) %>% pull(age) %>% mean(),
              dat %>% filter(nwhite==0, sis==1) %>% pull(age) %>% mean(),
              dat %>% filter(nwhite==1, sis==0) %>% pull(age) %>% mean(),
              dat %>% filter(nwhite==1, sis==1) %>% pull(age) %>% mean()),
        fem      =
            c(dat %>% filter(nwhite==0, sis==0) %>% pull(fem) %>% mean(),
              dat %>% filter(nwhite==0, sis==1) %>% pull(fem) %>% mean(),
              dat %>% filter(nwhite==1, sis==0) %>% pull(fem) %>% mean(),
              dat %>% filter(nwhite==1, sis==1) %>% pull(fem) %>% mean())
    )
    newdat <- cross.dummy.race.sis(newdat)
    
    newdat <- bind_rows(newdat %>% mutate(new = 0), 
                        newdat %>% mutate(new = 1))
    
    newlong <- bind_rows(newdat %>% select(-cd4.base) %>% mutate(post = 0),
                         newdat %>% select(-cd4.base) %>% mutate(post = 1))
    out <- list(newdat  = newdat,
                newlong = newlong)
}
```

## 2.2 Exploratory data analysis

### 2.2.1 Compare treatment arms w.r.t. baseline variables

Nothing particularly worrisome jumps out of the plots above.

Let’s compare treatment arms with regards to proportions in sex-by-race categories.

```
##   new       fem    nwhite F.nonwhite    F.white M.nonwhite   M.white
## 1   0 0.1428571 0.4681319 0.06687598 0.07598116  0.4012559 0.4558870
## 2   1 0.1736402 0.4728033 0.08209765 0.09154252  0.3907057 0.4356541
```

There are more females in the new treatment arm than in the old treatment arm in the restricted sample. Let’s compare these proportions to the original sample below.

```
##   new       fem    nwhite F.nonwhite    F.white M.nonwhite   M.white
## 1   0 0.1623489 0.4905009 0.07963226 0.08271661  0.4108686 0.4267825
## 2   1 0.1837088 0.4748700 0.08723782 0.09647102  0.3876322 0.4286590
```

This gender imbalance exists in the original data too. When weighting to the trial population, we might want to weight each group to the population separately.

### 2.2.2 Compare treatment arms w.r.t. post-treatment CD4 count

The plot above shows that there is an average treatment effect.

This plot, stratified by SIS status shows treatment effect more clearly. The effect is more pronounced for those with baseline SIS, but seems to also exist for those without SIS at baseline.

## 2.3 Models to estimate effects

Before searching for effect modification, we need to pick an effect scale. Since CD4 count is a non-negative variable, there is the question of whether we should consider CD4 change on an additive scale or on a multiplicative scale. The literature on CD4 count change as a result of antiretroviral therapy uses additive change, which is the convention we follow. While we have not seen a discussion or justification for this choice in the literature, it seems to make sense because there is generally a normal (i.e., no immune suppression) CD4 range, which puts a bound on CD4 increase on both the multiplicative and additive scales, but much more so on the multiplicative scale.

For data that include both baseline and post-treatment measures of the outcome, there are two modeling options: treating the baseline measure as a covariate to be controlled for, and modeling the combination of baseline and post-treatment measures using a two-level model. (We don’t consider the third, which is hard-coding the difference as the outcome, because it is equivalent to forcing the baseline measure to a have coefficient of 1 in a model for the post-treatment outcome.) With two measures of the outcome, these two modeling strategies are essentially equivalent [wish I knew of a cite somewhere for this], but the hierarchical model works for situations with more than one post-treatment measurement.

The analysis of real data is interesting and informative, but the ultimate purpose here is to use the results to generate a synthetic dataset with similar distribution. To generate such dataset, we can use either a two-level or a one-level as the basis. Therefore, we run both options in parallel below. The h. and f. in model names mean hierarchical (random intercepts) models and flat (one level) models, respectively.

### 2.3.1 SATE - hierarchical models

No covariates.

```
h.0 <- lmer(cd4 ~ new*post + (1|id), data = long)
```

Covariates having equal influences on CD4 count regardless of baseline or post-treatment, and regardless of treatment condition.

```
h.1 <- lmer(cd4 ~ age + fem + nwhite + sis + new*post + (1|id), data = long)
```

Covariates influencing CD4 count differently for the two treatment conditions.

```
h.1.new  <- lmer(cd4 ~ age + fem + nwhite + sis + 
                     age*new + fem*new + nwhite*new + sis*new + 
                     new*post + (1|id), data = long)
```

Covariates infuencing pre-to-post CD4 count change the same way for both conditions.

```
h.1.post  <- lmer(cd4 ~ age + fem + nwhite + sis + 
                      age*post + fem*post + nwhite*post + sis*post + 
                      new*post + (1|id), data = long)
```

Covariates influencing baseline CD4 count differently for the two treatment conditions, and influencing pre-to-post CD4 count change the same way for both conditions.

```
h.1.new.post <- lmer(cd4 ~ age + fem + nwhite + sis + 
                         age*new + fem*new + nwhite*new + sis*new + 
                         age*post + fem*post + nwhite*post + sis*post +
                         new*post + (1|id), data = long)
```

```
h.sate.ests <- rbind(
    contest(h.0, matrix(c(rep(0, 3), 1), nrow = 1), joint = FALSE),
    contest(h.1, matrix(c(rep(0, 7), 1), nrow = 1), joint = FALSE),
    contest(h.1.new,  matrix(c(rep(0, 11), 1), nrow = 1), joint = FALSE),
    contest(h.1.post, matrix(c(rep(0, 11), 1), nrow = 1), joint = FALSE),
    contest(h.1.new.post, matrix(c(rep(0, 15), 1), nrow = 1), joint = FALSE)
)
rownames(h.sate.ests) <- c("0", "1", "2.new", "2.post", "2.new.post")
h.sate.ests
```

```
##            Estimate Std. Error  df  t value    lower    upper     Pr(>|t|)
## 0          36.60212   4.415954 931 8.288610 27.93574 45.26849 3.973998e-16
## 1          36.60212   4.415954 931 8.288610 27.93574 45.26849 3.973999e-16
## 2.new      36.60212   4.415954 931 8.288610 27.93574 45.26849 3.973998e-16
## 2.post     35.93868   4.406401 927 8.156017 27.29100 44.58636 1.119636e-15
## 2.new.post 35.93868   4.406401 927 8.156017 27.29100 44.58636 1.119635e-15
```

### 2.3.2 SATE - flat models

```
f.0 <- lm(cd4.post ~ new, data = dat)
```

```
f.1.base <- lm(cd4.post ~ cd4.base + new, data = dat)
```

```
f.1.all <- lm(cd4.post ~ cd4.base + age + fem + nwhite + sis + new, data = dat)
```

```
f.sate.ests <- rbind(
    summary(f.0)$coef["new",],
    summary(f.1.base)$coef["new",],
    summary(f.1.all)$coef["new",]
)
rownames(f.sate.ests) <- c("0", "1.base", "1.all")
f.sate.ests
```

```
##        Estimate Std. Error  t value     Pr(>|t|)
## 0      43.19200   6.371486 6.778951 2.146816e-11
## 1.base 36.42762   4.421874 8.238050 5.905006e-16
## 1.all  36.29295   4.394502 8.258718 5.051788e-16
```

### 2.3.3 Effect modification - hierarchical models

```
h.2.age <- lmer(cd4 ~ age + fem + nwhite + sis + 
                         age*new + fem*new + nwhite*new + sis*new + 
                         age*post + fem*post + nwhite*post + sis*post +
                         new*post + age*new*post + (1|id), data = long)
summary(h.2.age)$coef[16:17,]
```

```
##                 Estimate Std. Error  df    t value   Pr(>|t|)
## new:post     38.89877368 19.9770611 926  1.9471720 0.05181611
## age:new:post -0.07485031  0.4926945 926 -0.1519203 0.87928285
```

```
h.2.sex <- lmer(cd4 ~ age + fem + nwhite + sis + 
                            age*new + fem*new + nwhite*new + sis*new + 
                            age*post + fem*post + nwhite*post + sis*post +
                            new*post + fem*new*post + (1|id), data = long)
summary(h.2.sex)$coef[16:17,]
```

```
##               Estimate Std. Error  df    t value     Pr(>|t|)
## new:post     36.638788   4.800149 926  7.6328437 5.704432e-14
## fem:new:post -4.481333  12.156607 926 -0.3686335 7.124852e-01
```

```
h.2.race <- lmer(cd4 ~ age + fem + nwhite + sis + 
                            age*new + fem*new + nwhite*new + sis*new + 
                            age*post + fem*post + nwhite*post + sis*post +
                            new*post + nwhite*new*post + (1|id), data = long)
summary(h.2.race)$coef[16:17,]
```

```
##                 Estimate Std. Error  df  t value     Pr(>|t|)
## new:post        30.06656   6.054431 926 4.966042 8.134296e-07
## nwhite:new:post 12.51004   8.850994 926 1.413405 1.578728e-01
```

```
h.2.sis <- lmer(cd4 ~ age + fem + nwhite + sis + 
                            age*new + fem*new + nwhite*new + sis*new + 
                            age*post + fem*post + nwhite*post + sis*post +
                            new*post + sis*new*post + (1|id), data = long)
summary(h.2.sis)$coef[16:17,]
```

```
##              Estimate Std. Error  df  t value     Pr(>|t|)
## new:post     30.01259   5.974867 926 5.023140 6.098415e-07
## sis:new:post 12.97436   8.841021 926 1.467519 1.425746e-01
```

It looks like race and SIS status are potential effect modifiers (with substantial coefficients, about 12, albeit not statistically significant). Let’s cross-classify these two variables and interact with treatment, to see if we get a stronger signal.

```
h.2.race.sis <- lmer(cd4 ~ age + fem + interaction(nwhite, sis) + 
                            age*new + fem*new + new*interaction(nwhite, sis) + 
                            age*post + fem*post + post*interaction(nwhite, sis) +
                            new*post + new*post*interaction(nwhite, sis) + (1|id),
                     data = long)
summary(h.2.race.sis)$coef[19:22,]
```

```
##                                      Estimate Std. Error  df  t value    Pr(>|t|)
## new:post                             21.32591   7.951362 923 2.682045 0.007448076
## interaction(nwhite, sis)1.0:new:post 20.01050  12.102804 923 1.653378 0.098594089
## interaction(nwhite, sis)0.1:new:post 20.67795  12.266800 923 1.685684 0.092194686
## interaction(nwhite, sis)1.1:new:post 22.51718  12.084005 923 1.863387 0.062725234
```

The signal is clearer when we collapse the three groups that are not white without SIS.

```
h.2.race.sis.b <- lmer(cd4 ~ age + fem + interaction(nwhite, sis) + 
                            age*new + fem*new + new*interaction(nwhite, sis) + 
                            age*post + fem*post + post*interaction(nwhite, sis) +
                            new*post + new*post*I(!(nwhite==0 & sis==0)) +
                           (1|id), data = long)
summary(h.2.race.sis.b)$coef[19:20,]
```

```
##                                           Estimate Std. Error  df  t value    Pr(>|t|)
## new:post                                  21.32348   7.942893 925 2.684599 0.007391571
## new:post:I(!(nwhite == 0 & sis == 0))TRUE 21.07678   9.552352 925 2.206449 0.027597620
```

### 2.3.4 Effect modification – flat models

```
f.2.age <- lm(cd4.post ~ cd4.base + age + fem + nwhite + sis + age*new, data = dat)
summary(f.2.age)$coef[7:8,]
```

```
##            Estimate Std. Error    t value   Pr(>|t|)
## new     39.97995831 19.9178569  2.0072420 0.04501442
## age:new -0.09322224  0.4911793 -0.1897927 0.84951322
```

```
f.2.sex <- lm(cd4.post ~ cd4.base + age + fem + nwhite + sis + fem*new, data = dat)
summary(f.2.sex)$coef[7:8,]
```

```
##          Estimate Std. Error    t value     Pr(>|t|)
## new     37.103506   4.788064  7.7491673 2.429227e-14
## fem:new -5.180234  12.120674 -0.4273883 6.691960e-01
```

```
f.2.race <- lm(cd4.post ~ cd4.base + age + fem + nwhite + sis + nwhite*new, data = dat)
summary(f.2.race)$coef[7:8,]
```

```
##            Estimate Std. Error  t value     Pr(>|t|)
## new        30.33759   6.035877 5.026210 6.005363e-07
## nwhite:new 12.69058   8.822859 1.438375 1.506661e-01
```

```
f.2.sis <- lm(cd4.post ~ cd4.base + age + fem + nwhite + sis + sis*new, data = dat)
summary(f.2.sis)$coef[7:8,]
```

```
##         Estimate Std. Error  t value     Pr(>|t|)
## new     30.77581   5.964222 5.160071 3.021130e-07
## sis:new 12.06281   8.821463 1.367438 1.718201e-01
```

```
f.2.race.sis <- lm(cd4.post ~ cd4.base + age + fem + interaction(nwhite, sis) + new + new*interaction(nwhite, sis), data = dat)
summary(f.2.race.sis)$coef[8:11,]
```

```
##                                 Estimate Std. Error  t value    Pr(>|t|)
## new                             22.21259   7.935238 2.799234 0.005229323
## interaction(nwhite, sis)1.0:new 19.73532  12.067190 1.635453 0.102295426
## interaction(nwhite, sis)0.1:new 19.25027  12.242971 1.572353 0.116211791
## interaction(nwhite, sis)1.1:new 21.88873  12.050476 1.816420 0.069630524
```

```
f.2.race.sis.b <- lm(cd4.post ~ cd4.base + age + fem + interaction(nwhite, sis) + new + new*I(!(nwhite==0 & sis==0)), data = dat)
summary(f.2.race.sis.b)$coef[8:9,]
```

```
##                                      Estimate Std. Error  t value   Pr(>|t|)
## new                                  22.21134   7.926833 2.802044 0.00518414
## new:I(!(nwhite == 0 & sis == 0))TRUE 20.30959   9.528612 2.131432 0.03331693
```

### 2.3.5 Visualizing effect modification

Here we use synthetic observations with group-specific mean baseline covariates.

```
newlong <- construct.newdata(dat)$newlong
newdat  <- construct.newdata(dat)$newdat

p1 <- newlong %>%
    bind_cols(cd4.pred = predict(h.2.race.sis, newdata = newlong, re.form = NA)) %>%
    mutate(new  = factor(new,  labels = c("old", "new")),
           post = factor(post, labels = c("pre", "post"))) %>%
    ggplot(aes(x = nwhite.sis.plot, 
               y = cd4.pred,
               color = new,
               shape = post)) +
    geom_point() +
    labs(x = "",
         y = "Predicted mean CD4 count",
         color = "Treatment",
         shape = "Time",
         title = "Predictions from model h.2.race.sis") +
    theme_bw() +
    ylim(0, 250)

p2 <- newdat %>% 
    bind_cols(cd4.post.pred = predict(f.2.race.sis, newdata = newdat)) %>%
    mutate(new = factor(new, labels = c("old", "new"))) %>%
    ggplot(aes(x = nwhite.sis.plot, 
               y = cd4.post.pred,
               color = new)) +
    geom_point() +
    labs(x = "",
         y = "Predicted mean post-treatment CD4 count",
         color = "Treatment",
         title = "Predictions from model f.2.race.sis") +
    theme_bw() +
    ylim(0, 250)

gridExtra::grid.arrange(p1, p2, ncol = 2)
```

These predicted mean CD4 counts are consistent between h and f models.

## 2.4 Further fiddling in consideration of synthetic data generation

In making synthetic data, we will need to

- generate/borrow data on baseline covariates
- use a model (any of the four models above would be appropriate) to generate conditional means for the outcome
- generate the random elements (error terms, random effects)

With the baseline covariates, it is safe to borrow the categorical variables (fem, nwhite, sis). These form 8 patterns with plenty of observations in each, so that does not confer any risk of identifying any individual.

We do not want to borrow the continuous covariate age. Instead, we want to generate a new variable age that only preserves the range of age in the data.

To avoid causing substantial distortion to the CD4 count distributions, let’s consider the role of variable age in the models we have fit.

```
summary(h.2.race.sis)$coef["age",]; summary(f.2.race.sis)$coef["age",]
```

```
##     Estimate   Std. Error           df      t value     Pr(>|t|) 
## 9.541396e-02 2.980936e-01 1.294321e+03 3.200805e-01 7.489589e-01
```

```
##   Estimate Std. Error    t value   Pr(>|t|) 
## -0.1437725  0.2484484 -0.5786814  0.5629455
```

Age has a very statistically non-sig coefficient in these models. So let’s refit these models without age. And for simplicity, let’s also leave out any associations with CD4 count with sex.

```
h.3 <- lmer(cd4 ~ interaction(nwhite, sis) + 
                new*interaction(nwhite, sis) + 
                post*interaction(nwhite, sis) +
                new*post + new*post*interaction(nwhite, sis) + (1|id),
            data = long)
f.3 <- lm(cd4.post ~ cd4.base + interaction(nwhite, sis) + 
              new + new*interaction(nwhite, sis), data = dat)
summary(h.3)$coef; summary(f.3)$coef
```

```
##                                         Estimate Std. Error       df     t value      Pr(>|t|)
## (Intercept)                           128.703008   5.317539 1593.132  24.2034901 1.808421e-110
## interaction(nwhite, sis)1.0             4.551709   7.984665 1593.132   0.5700564  5.687198e-01
## interaction(nwhite, sis)0.1          -100.384965   7.923282 1593.132 -12.6696185  4.048465e-35
## interaction(nwhite, sis)1.1          -106.137587   7.963875 1593.132 -13.3273799  1.701035e-38
## new                                     6.649806   7.259242 1593.132   0.9160469  3.597809e-01
## post                                   44.342105   5.817590  925.000   7.6220744  6.176508e-14
## interaction(nwhite, sis)1.0:new        -2.417567  10.994422 1593.132  -0.2198903  8.259848e-01
## interaction(nwhite, sis)0.1:new       -10.637917  11.205965 1593.132  -0.9493084  3.426077e-01
## interaction(nwhite, sis)1.1:new        -4.788800  11.032869 1593.132  -0.4340485  6.643120e-01
## interaction(nwhite, sis)1.0:post       -8.747766   8.735528  925.000  -1.0014010  3.168949e-01
## interaction(nwhite, sis)0.1:post      -25.329873   8.668372  925.000  -2.9221026  3.561386e-03
## interaction(nwhite, sis)1.1:post      -22.477619   8.712782  925.000  -2.5798441  1.003776e-02
## new:post                               21.038847   7.941887  925.000   2.6490992  8.208256e-03
## interaction(nwhite, sis)1.0:new:post   21.271161  12.028316  925.000   1.7684238  7.731963e-02
## interaction(nwhite, sis)0.1:new:post   20.710825  12.259752  925.000   1.6893348  9.149229e-02
## interaction(nwhite, sis)1.1:new:post   22.931502  12.070378  925.000   1.8998163  5.776801e-02
```

```
##                                    Estimate  Std. Error    t value     Pr(>|t|)
## (Intercept)                      61.7503641  8.91748749  6.9246370 8.177780e-12
## cd4.base                          0.8647409  0.05262936 16.4307689 2.040327e-53
## interaction(nwhite, sis)1.0      -8.1321053  8.71247462 -0.9333864 3.508643e-01
## interaction(nwhite, sis)0.1     -38.9078574 10.12917907 -3.8411659 1.308096e-04
## interaction(nwhite, sis)1.1     -36.8336986 10.32754679 -3.5665487 3.802797e-04
## new                              21.9382942  7.92566461  2.7680069 5.752812e-03
## interaction(nwhite, sis)1.0:new  20.9441631 11.99271299  1.7464074 8.107248e-02
## interaction(nwhite, sis)0.1:new  19.2719496 12.23559149  1.5750730 1.155819e-01
## interaction(nwhite, sis)1.1:new  22.2837727 12.03661222  1.8513326 6.444063e-02
```

There is little change when leaving out these two variables. Let’s look at the predicted means.

Regarding the last point (generation of random elements), theoretically we could generate these from normal distributions with model-estimated variances. But the normality assumption seldom holds. Let’s look at the distribution of residuals.

Let’s look at the residuals.

```
long <- long %>%
    bind_cols(cd4.pred = predict(h.3, re.form = NULL)) %>%
    mutate(residual = cd4 - cd4.pred)

dat <- dat %>%
    bind_cols(cd4.post.pred = predict(f.3)) %>%
    mutate(residual = cd4.post - cd4.post.pred)
```

The distribution of residuals varies by group and is not normal. This suggets we may do better drawing from existing residuals than generating normal errors.

The residuals in cd4.post from the h model have slightly smaller variance than those from the f model, but they are actually quite close.

Before moving on, let’s take stock of what we have.

```
names(dat)
```

```
##  [1] "id"            "new"           "cd4.base"      "cd4.post"      "age"           "fem"          
##  [7] "nwhite"        "sis"           "cd4.post.pred" "residual"
```

```
names(long)
```

```
##  [1] "id"       "new"      "age"      "fem"      "nwhite"   "sis"      "post"     "cd4"     
##  [9] "cd4.pred" "residual"
```

The predicted values and residuals in the datasets above are from models h.3 and f.3, respectively.

# 3 Make synthetic trial data

## 3.1 Methods

3 steps for making synthetic data based on model h.3

- Obtain sex, race, SIS status, treatment, and the model-predicted conditional mean of CD4 count. De-id the data and generate a fake id variable.
- Get residual pairs and permute them, and assign them as error terms.
- Draw random age values from the estimated age distribution.

4 steps for making synthetic data based on model f.3

- Obtain sex, race, SIS status, treatment from the real data, and a heavily jittered variable baseline CD4 count.
- Based on these baseline variables, obtain the model predicted mean post-treatment CD4 count.
- Get residuals and permute them, and assign them as error terms.
- Draw random age values from the estimated age distribution.

Two functions for making synthetic trial data based on models h.3 and f.3 for a given set of seeds.

```
get.hmodel.synth <- function(seed, real.long = long) {
    set.seed(seed)
    
    # step 1
    synth <- real.long %>% 
        select(id, post, new, sis, fem, nwhite, cd4.pred) %>%
        spread(post, cd4.pred) %>%
        select(-id) %>%
        rename(mean.base = "0", mean.post = "1") %>%
        sample_frac(1) %>%
        mutate(id = 1:n()) %>%
        arrange(new, sis)
    
    # step 2
    resids <- real.long %>% 
        select(id, post, new, sis, residual) %>%
        spread(post, residual) %>%
        select(-id) %>%
        rename(resid.base = "0", resid.post = "1") %>%
        group_by(new, sis) %>%
        sample_frac(1) %>%
        arrange(new, sis)
    
    synth <- synth %>% 
        bind_cols(resids %>% select(new, sis, resid.base, resid.post)) %>%
        mutate(cd4.base = round(mean.base + resid.base),
               cd4.post = round(mean.post + resid.post),
               cd4.base = ifelse(cd4.base < 0, 0, cd4.base),
               cd4.post = ifelse(cd4.post < 0, 0, cd4.post)) %>%
        select(id, fem, nwhite, sis, new, cd4.base, cd4.post) 
    
    # step 3
    synth <- synth %>%
        bind_cols(age = round(rnorm(n = nrow(dat), 
                                    mean = sample(dat$age, replace = TRUE), 
                                    sd = density(dat$age)$bw)))
    synth
}
```

```
get.fmodel.synth <- function(seed, real.dat = dat) {
    set.seed(seed)
    
    # step 1
    synth <- dat %>%
        select(new, sis, fem, nwhite, cd4.base) %>%
        sample_frac(1) %>%
        mutate(id = 1:n(),
               cd4.base = ifelse(sis == 1, 
                                 round(jitter(cd4.base, amount = 4)),
                                 round(jitter(cd4.base, amount = 8))),
               cd4.base = ifelse(cd4.base < 0, 0, cd4.base)) %>%
        arrange(new, sis)
    
    # step 2
    synth <- synth %>% bind_cols(mean.post = predict(f.3, newdata = synth))
    
    # step 3
    resids <- dat %>% 
        select(new, sis, residual) %>%
        group_by(new, sis) %>%
        sample_frac(1) %>%
        arrange(new, sis)
    
    synth <- synth %>% 
        bind_cols(resid.post = resids$residual) %>%
        mutate(cd4.post = round(mean.post + resid.post),
               cd4.post = ifelse(cd4.post < 0, 0, cd4.post)) %>%
        select(id, fem, nwhite, sis, new, cd4.base, cd4.post) 
    
    # step 4
    synth <- synth %>%
        bind_cols(age = round(rnorm(n = nrow(dat), 
                                    mean = sample(dat$age, replace = TRUE), 
                                    sd = density(dat$age)$bw)))
    synth
}
```

## 3.2 Twodatasets based on the hierarchical model and the flat model

The seeds in h.seed and f.seed are hidden. Results vary a lot by seeds. What are shown here are from one of infinitely many sets of seeds.

```
sdat.h <- get.hmodel.synth(seed = h.seed)
sdat.f <- get.fmodel.synth(seed = f.seed)
```

Let’s compare these two synthetic datasets and the original real data with respect to the distributions of CD4 counts.

```
## # A tibble: 3 x 7
##   data   cd4.base.mean cd4.post.mean cd4.base.sd cd4.post.sd cd4.base.zeros cd4.post.zeros
##   <chr>          <dbl>         <dbl>       <dbl>       <dbl>          <int>          <int>
## 1 dat             84.1          134.        68.4        99.6             18             14
## 2 sdat.f          84.0          134.        68.5       101.              21              7
## 3 sdat.h          85.1          134.        65.7        87.1             56              2
```

The dataset based on the h model has a lot of zeros in baseline CD4, because we have a truncate the CD4 distribution at zero. This is not desirable.

Let’s have a quick peak at modelfit to these datasets. (This is just a quick peak; we will do a proper analysis later.)

```
slong.h <- sdat.h %>%
    gather("post", "cd4", -c(id, new, age, fem, nwhite, sis)) %>%
    mutate(post = as.integer(post == "cd4.post"))

slong.f <- sdat.f %>%
    gather("post", "cd4", -c(id, new, age, fem, nwhite, sis)) %>%
    mutate(post = as.integer(post == "cd4.post"))

h.h <- lmer(cd4 ~ age + fem + interaction(nwhite, sis) +
                age*new + fem*new + new*interaction(nwhite, sis) +
                age*post + fem*post + post*interaction(nwhite, sis) +
                new*post + new*post*interaction(nwhite, sis) + (1|id),
            data = slong.h)
h.f <- lmer(cd4 ~ age + fem + interaction(nwhite, sis) +
                age*new + fem*new + new*interaction(nwhite, sis) +
                age*post + fem*post + post*interaction(nwhite, sis) +
                new*post + new*post*interaction(nwhite, sis) + (1|id),
            data = slong.f)

summary(h.h)$coef[19:22,]; summary(h.f)$coef[19:22,]
```

```
##                                       Estimate Std. Error  df   t value     Pr(>|t|)
## new:post                             27.546028    7.59346 923 3.6275990 0.0003017532
## interaction(nwhite, sis)1.0:new:post  5.402486   11.55941 923 0.4673669 0.6403477486
## interaction(nwhite, sis)0.1:new:post  7.460379   11.71010 923 0.6370892 0.5242247192
## interaction(nwhite, sis)1.1:new:post 17.268562   11.53578 923 1.4969564 0.1347464601
```

```
##                                      Estimate Std. Error  df  t value   Pr(>|t|)
## new:post                             19.72150   7.862622 923 2.508260 0.01230331
## interaction(nwhite, sis)1.0:new:post 23.44112  11.979436 923 1.956780 0.05067447
## interaction(nwhite, sis)0.1:new:post 24.93563  12.135934 923 2.054694 0.04018965
## interaction(nwhite, sis)1.1:new:post 21.43789  11.956149 923 1.793043 0.07329338
```

## 3.3 Finalize

We pick the f dataset above. We’ll write the data out to submit with the paper.

```
sdat <- get.fmodel.synth(seed = f.seed)

slong <- sdat %>%
    gather("post", "cd4", -c(id, new, age, fem, nwhite, sis)) %>%
    mutate(post = as.integer(post == "cd4.post"))

glimpse(slong, width = 60)
```

```
## Observations: 1,866
## Variables: 8
## $ id     <int> 1, 4, 12, 13, 17, 21, 22, 26, 30, 37, 40...
## $ fem    <int> 0, 0, 0, 0, 0, 0, 0, 0, 0, 0, 0, 0, 1, 0...
## $ nwhite <int> 1, 1, 1, 1, 0, 1, 1, 0, 0, 1, 0, 1, 1, 0...
## $ sis    <int> 0, 0, 0, 0, 0, 0, 0, 0, 0, 0, 0, 0, 0, 0...
## $ new    <int> 0, 0, 0, 0, 0, 0, 0, 0, 0, 0, 0, 0, 0, 0...
## $ age    <dbl> 40, 48, 37, 38, 40, 55, 41, 34, 49, 27, ...
## $ post   <int> 0, 0, 0, 0, 0, 0, 0, 0, 0, 0, 0, 0, 0, 0...
## $ cd4    <dbl> 112, 98, 124, 59, 147, 180, 108, 87, 91,...
```

```
write_csv(slong, here::here("data", "synthetictrial.csv"))
```

The data file slong.rds can be read using function readRDS().

# 4 Synthetic trial data analysis

## 4.1 Estimate SATE

No covariates

```
s.0 <- lmer(cd4 ~ new*post + (1|id), data = slong)
```

Covariates having equal influences on CD4 count regardless of baseline or post-treatment, and regardless of treatment condition.

```
s.1 <- lmer(cd4 ~ age + fem + nwhite + sis + new*post + (1|id), data = slong)
```

Covariates influencing CD4 count differently for the two treatment conditions.

```
s.1.new  <- lmer(cd4 ~ age + fem + nwhite + sis + 
                     age*new + fem*new + nwhite*new + sis*new + 
                     new*post + (1|id), data = slong)
```

Covariates infuencing pre-to-post CD4 count change the same way for both conditions.

```
s.1.post  <- lmer(cd4 ~ age + fem + nwhite + sis + 
                      age*post + fem*post + nwhite*post + sis*post + 
                      new*post + (1|id), data = slong)
```

Covariates influencing baseline CD4 count differently for the two treatment conditions, and influencing pre-to-post CD4 count change the same way for both conditions.

```
s.1.new.post <- lmer(cd4 ~ age + fem + nwhite + sis + 
                         age*new + fem*new + nwhite*new + sis*new + 
                         age*post + fem*post + nwhite*post + sis*post +
                         new*post + (1|id), data = slong)
```

```
sate.ests <- rbind(
    contest(s.0, matrix(c(rep(0, 3), 1), nrow = 1), joint = FALSE),
    contest(s.1, matrix(c(rep(0, 7), 1), nrow = 1), joint = FALSE),
    contest(s.1.new,  matrix(c(rep(0, 11), 1), nrow = 1), joint = FALSE),
    contest(s.1.post, matrix(c(rep(0, 11), 1), nrow = 1), joint = FALSE),
    contest(s.1.new.post, matrix(c(rep(0, 15), 1), nrow = 1), joint = FALSE)
)
rownames(sate.ests) <- c("0", "1", "1.new", "1.post", "1.new.post")
sate.ests
```

```
##            Estimate Std. Error  df  t value    lower    upper     Pr(>|t|)
## 0          36.55986   4.383802 931 8.339761 27.95658 45.16314 2.660009e-16
## 1          36.55986   4.383802 931 8.339761 27.95658 45.16314 2.660009e-16
## 1.new      36.55986   4.383802 931 8.339761 27.95658 45.16314 2.660013e-16
## 1.post     35.88346   4.361957 927 8.226460 27.32301 44.44392 6.486639e-16
## 1.new.post 35.88346   4.361957 927 8.226460 27.32301 44.44392 6.486635e-16
```

```
m.sate <- s.1.new.post
SATE <- sate.ests["1.new.post",]
SATE
```

```
##            Estimate Std. Error  df t value    lower    upper     Pr(>|t|)
## 1.new.post 35.88346   4.361957 927 8.22646 27.32301 44.44392 6.486635e-16
```

## 4.2 Search for effect modifications

Interaction with one covariate at a time.

```
s.2.age <- lmer(cd4 ~ age + fem + nwhite + sis + 
                            age*new + fem*new + nwhite*new + sis*new + 
                            age*post + fem*post + nwhite*post + sis*post +
                            new*post + age*new*post + (1|id), data = slong)
summary(s.2.age)$coef[16:17,]
```

```
##                Estimate Std. Error  df   t value     Pr(>|t|)
## new:post     66.2106057 19.8738125 926  3.331550 0.0008978053
## age:new:post -0.7676332  0.4907944 926 -1.564063 0.1181445437
```

```
s.2.sex <- lmer(cd4 ~ age + fem + nwhite + sis + 
                            age*new + fem*new + nwhite*new + sis*new + 
                            age*post + fem*post + nwhite*post + sis*post +
                            new*post + fem*new*post + (1|id), data = slong)
summary(s.2.sex)$coef[16:17,]
```

```
##               Estimate Std. Error  df   t value     Pr(>|t|)
## new:post     34.324406   4.750698 926 7.2251284 1.045487e-12
## fem:new:post  9.973967  12.029992 926 0.8290918 4.072661e-01
```

```
s.2.race <- lmer(cd4 ~ age + fem + nwhite + sis + 
                            age*new + fem*new + nwhite*new + sis*new + 
                            age*post + fem*post + nwhite*post + sis*post +
                            new*post + nwhite*new*post + (1|id), data = slong)
summary(s.2.race)$coef[16:17,]
```

```
##                 Estimate Std. Error  df  t value     Pr(>|t|)
## new:post        30.35849   5.991056 926 5.067302 4.870728e-07
## nwhite:new:post 11.77988   8.760551 926 1.344650 1.790675e-01
```

```
s.2.sis <- lmer(cd4 ~ age + fem + nwhite + sis + 
                            age*new + fem*new + nwhite*new + sis*new + 
                            age*post + fem*post + nwhite*post + sis*post +
                            new*post + sis*new*post + (1|id), data = slong)
summary(s.2.sis)$coef[16:17,]
```

```
##              Estimate Std. Error  df  t value     Pr(>|t|)
## new:post     29.89819   5.921342 926 5.049225 5.341286e-07
## sis:new:post 13.09494   8.768058 926 1.493482 1.356517e-01
```

Interaction with cross-classifications

```
s.3.sex.race <- lmer(cd4 ~ age + sis + interaction(fem, nwhite) +
                         new + age*new + sis*new + new*interaction(fem, nwhite) +
                         post + age*post + sis*post + post*interaction(fem, nwhite) +
                         new*post + new*post*interaction(fem, nwhite) + (1|id),
                     data = slong)
summary(s.3.sex.race)$coef[19:22,]
```

```
##                                      Estimate Std. Error  df   t value     Pr(>|t|)
## new:post                             27.48889   6.279561 923 4.3775175 1.337542e-05
## interaction(fem, nwhite)1.0:new:post 34.19427  21.228083 923 1.6108037 1.075644e-01
## interaction(fem, nwhite)0.1:new:post 16.09933   9.621761 923 1.6732211 9.462263e-02
## interaction(fem, nwhite)1.1:new:post 11.04217  14.793243 923 0.7464332 4.555958e-01
```

```
s.3.sex.sis <- lmer(cd4 ~ age + nwhite + interaction(fem, sis) +
                         new + age*new + nwhite*new + new*interaction(fem, sis) +
                         post + age*post + nwhite*post + post*interaction(fem, sis) +
                         new*post + new*post*interaction(fem, sis) + (1|id),
                     data = slong)
summary(s.3.sex.sis)$coef[19:22,]
```

```
##                                    Estimate Std. Error  df   t value     Pr(>|t|)
## new:post                          28.527571   6.454427 923 4.4198457 1.105001e-05
## interaction(fem, sis)1.0:new:post  8.873224  16.488192 923 0.5381563 5.905989e-01
## interaction(fem, sis)0.1:new:post 12.677687   9.557645 923 1.3264446 1.850206e-01
## interaction(fem, sis)1.1:new:post 24.066481  17.522574 923 1.3734558 1.699442e-01
```

```
s.3.race.sis <- lmer(cd4 ~ age + fem + interaction(nwhite, sis) +
                         new + age*new + fem*new + new*interaction(nwhite, sis) +
                         post + age*post + fem*post + post*interaction(nwhite, sis) +
                         new*post + new*post*interaction(nwhite, sis) + (1|id),
                     data = slong)
summary(s.3.race.sis)$coef[19:22,]
```

```
##                                      Estimate Std. Error  df  t value   Pr(>|t|)
## new:post                             19.72150   7.862622 923 2.508260 0.01230331
## interaction(nwhite, sis)1.0:new:post 23.44112  11.979436 923 1.956780 0.05067447
## interaction(nwhite, sis)0.1:new:post 24.93563  12.135934 923 2.054694 0.04018965
## interaction(nwhite, sis)1.1:new:post 21.43789  11.956149 923 1.793043 0.07329338
```

Let’s visualize the results.

```
snewlong <- construct.newdata(sdat)$newlong

snewlong %>%
    bind_cols(cd4.pred = predict(s.3.race.sis,
                                 newdata = snewlong, re.form = NA)) %>%
    mutate(new  = factor(new,  labels = c("old", "new")),
           post = factor(post, labels = c("pre", "post"))) %>%
    ggplot(aes(x = nwhite.sis.plot, 
               y = cd4.pred,
               color = new,
               shape = post)) +
    geom_point() +
    labs(x = "",
         y = "Predicted mean CD4 count",
         color = "Treatment",
         shape = "Time",
         title = "Predictions from s.3.race.sis model") +
    theme_bw() +
    ylim(0, 250)
```

```
s.3.race.sis.effs <- 
    contest(s.3.race.sis, cbind(matrix(0, ncol = 18, nrow = 4),
                                matrix(c(1, 0, 0, 0,
                                         1, 1, 0, 0,
                                         1, 0, 1, 0,
                                         1, 0, 0, 1), byrow = TRUE, ncol = 4)),
            joint = FALSE, check_estimability = TRUE)

rownames(s.3.race.sis.effs) <-
    c("white.nosis", "nonwhite.nosis", "white.sis", "nonwhite.sis")
s.3.race.sis.effs
```

```
##                Estimate Std. Error  df  t value     lower    upper     Pr(>|t|)
## white.nosis    19.72150   7.862622 923 2.508260  4.290806 35.15219 1.230331e-02
## nonwhite.nosis 43.16262   9.016177 923 4.787242 25.468037 60.85721 1.968485e-06
## white.sis      44.65712   9.246912 923 4.829409 26.509712 62.80453 1.602462e-06
## nonwhite.sis   41.15939   9.003218 923 4.571631 23.490234 58.82854 5.497825e-06
```

```
m.effmod <- s.3.race.sis
```

```
png(filename = here::here("Model.png"),
    width = 8.5, height = 4, units = "in", res = 2400)
p1 <- snewlong %>%
    bind_cols(cd4.pred = predict(m.effmod,
                                 newdata = snewlong, re.form = NA)) %>%
    mutate(new  = factor(new,  labels = c("old", "new")),
           post = factor(post, labels = c("pre", "post"))) %>%
    ggplot(aes(x = nwhite.sis.plot, 
               y = cd4.pred,
               color = new,
               shape = post)) +
    geom_point() +
    labs(x = "",
         y = "Predicted mean CD4 count",
         color = "Treatment",
         shape = "Time",
         title = "Model-predicted mean CD4 counts") +
    theme_bw() +
    ylim(0, 250)
p2 <- s.3.race.sis.effs %>% 
    as_tibble(rownames = "group") %>%
    mutate(group = factor(group, levels = c("white.nosis",
                                            "nonwhite.nosis",
                                            "white.sis",
                                            "nonwhite.sis"))) %>%
    ggplot(aes(x = group, y = Estimate, min = lower, max = upper)) +
    geom_point(shape = 15) +
    geom_errorbar(width = 0) +
    labs(x = "",
         y = "Treatment effect",
         title = "Model-estimated treatment effects") +
    theme_bw()

gridExtra::grid.arrange(gridExtra::arrangeGrob(p1, p2, ncol = 2, widths = c(1.3, 1)))
dev.off()
```

```
## png 
##   2
```

# 5 Population data

Input the summary target population dataset that captures the joint distribution several characteristics in the form of frequencies of patterns of characteristics.

```
pop <- read_csv(here::here("data", "us.csv"), col_types = cols())

pop <- pop %>% 
    mutate(fem = as.integer(1 - male),
           nwhite = as.integer(1 - white)) %>%
    select(agegp, fem, nwhite, n) %>%
    uncount(weights = n)
glimpse(pop)
```

```
## Observations: 54,220
## Variables: 3
## $ agegp  <int> 1, 1, 1, 1, 1, 1, 1, 1, 1, 1, 1, 1, 1, 1, 1, 1, 1, 1, 1, 1, 1, 1, 1, 1, 1, 1, 1,...
## $ fem    <int> 1, 1, 1, 1, 1, 1, 1, 1, 1, 1, 1, 1, 1, 1, 1, 1, 1, 1, 1, 1, 1, 1, 1, 1, 1, 1, 1,...
## $ nwhite <int> 1, 1, 1, 1, 1, 1, 1, 1, 1, 1, 1, 1, 1, 1, 1, 1, 1, 1, 1, 1, 1, 1, 1, 1, 1, 1, 1,...
```

```
table(pop$agegp)
```

```
## 
##     1     2     3     4 
## 18500 16740 13370  5610
```

# 6 Outcome-model-based sensitivity analysis

## 6.1 Trial-population covariates balance

```
sdat <- sdat %>% 
    mutate(agegp   = ifelse(age <= 29, 1,
                            ifelse(age <= 39, 2,
                                   ifelse(age <= 49, 3, 4))))
stack <- 
    bind_rows(pop %>% 
                  mutate(id = NA,
                         cd4.base = NA,
                         cd4.post = NA,
                         age = NA,
                         sis = NA,
                         new = NA,
                         sample = 0) %>%
                  select(id, cd4.base, cd4.post,
                         age, agegp, fem, nwhite, sis, new, sample),
              sdat %>% 
                  mutate(sample = 1) %>%
                  select(id, cd4.base, cd4.post, 
                         age, agegp, fem, nwhite, sis, new, sample)) %>%
    mutate(agegp = factor(agegp))

glimpse(stack)
```

```
## Observations: 55,153
## Variables: 10
## $ id       <int> NA, NA, NA, NA, NA, NA, NA, NA, NA, NA, NA, NA, NA, NA, NA, NA, NA, NA, NA, NA...
## $ cd4.base <dbl> NA, NA, NA, NA, NA, NA, NA, NA, NA, NA, NA, NA, NA, NA, NA, NA, NA, NA, NA, NA...
## $ cd4.post <dbl> NA, NA, NA, NA, NA, NA, NA, NA, NA, NA, NA, NA, NA, NA, NA, NA, NA, NA, NA, NA...
## $ age      <dbl> NA, NA, NA, NA, NA, NA, NA, NA, NA, NA, NA, NA, NA, NA, NA, NA, NA, NA, NA, NA...
## $ agegp    <fct> 1, 1, 1, 1, 1, 1, 1, 1, 1, 1, 1, 1, 1, 1, 1, 1, 1, 1, 1, 1, 1, 1, 1, 1, 1, 1, ...
## $ fem      <int> 1, 1, 1, 1, 1, 1, 1, 1, 1, 1, 1, 1, 1, 1, 1, 1, 1, 1, 1, 1, 1, 1, 1, 1, 1, 1, ...
## $ nwhite   <int> 1, 1, 1, 1, 1, 1, 1, 1, 1, 1, 1, 1, 1, 1, 1, 1, 1, 1, 1, 1, 1, 1, 1, 1, 1, 1, ...
## $ sis      <int> NA, NA, NA, NA, NA, NA, NA, NA, NA, NA, NA, NA, NA, NA, NA, NA, NA, NA, NA, NA...
## $ new      <int> NA, NA, NA, NA, NA, NA, NA, NA, NA, NA, NA, NA, NA, NA, NA, NA, NA, NA, NA, NA...
## $ sample   <dbl> 0, 0, 0, 0, 0, 0, 0, 0, 0, 0, 0, 0, 0, 0, 0, 0, 0, 0, 0, 0, 0, 0, 0, 0, 0, 0, ...
```

```
stack %>%
    group_by(sample) %>%
    summarize(age     = mean(age),
              agegp.1 = mean(agegp == 1),
              agegp.2 = mean(agegp == 2),
              agegp.3 = mean(agegp == 3),
              agegp.4 = mean(agegp == 4),
              female  = mean(fem),
              male    = 1 - female,
              nwhite  = mean(nwhite),
              white   = 1 - nwhite,
              sis     = mean(sis))
```

```
## # A tibble: 2 x 11
##   sample   age agegp.1 agegp.2 agegp.3 agegp.4 female  male nwhite white    sis
##    <dbl> <dbl>   <dbl>   <dbl>   <dbl>   <dbl>  <dbl> <dbl>  <dbl> <dbl>  <dbl>
## 1      0  NA     0.341   0.309   0.247   0.103  0.266 0.734  0.639 0.361 NA    
## 2      1  39.5   0.107   0.421   0.348   0.123  0.159 0.841  0.471 0.529  0.456
```

## 6.2 Estimate TATE

For sensitivity analysis, we need a plausible range for the proportion of people in the target population who have SIS. We specify this to be a wide range of (.20, .60).

```
pop.mean.nwhite <- mean(pop$nwhite)
pop.mean.sis    <- c(.2, .6)

TATE <- contest(m.effmod, cbind(rbind(c(rep(0, 18), 1),
                                      c(rep(0, 18), 1)),
                                pop.mean.nwhite * (1 - pop.mean.sis),
                                (1 - pop.mean.nwhite) * pop.mean.sis,
                                pop.mean.nwhite * pop.mean.sis),
                joint = FALSE, check_estimability = TRUE)

TATE <- cbind(pop.mean.sis = pop.mean.sis, TATE)
TATE
```

```
##   pop.mean.sis Estimate Std. Error  df  t value    lower    upper     Pr(>|t|)
## 1          0.2 36.24252   5.299374 923 6.839019 25.84230 46.64274 1.449615e-11
## 2          0.6 39.33251   4.741525 923 8.295329 30.02709 48.63793 3.809404e-16
```

# 7 Weighted outcome-model-based sensitivity analysis

## 7.1 Weighting trial sample to mimic target population

Under 6.1 above, we see that the target population and the trial sample differ substantially with respect to the distribution of the variables that are observed in both.

Note that there are also differences between the new and old treatment arms, especially in sex.

```
sdat %>%
    group_by(new) %>%
    summarize(age     = mean(age),
              agegp.1 = mean(agegp == 1),
              agegp.2 = mean(agegp == 2),
              agegp.3 = mean(agegp == 3),
              agegp.4 = mean(agegp == 4),
              female  = mean(fem),
              male    = 1 - female,
              nwhite  = mean(nwhite),
              white   = 1 - nwhite,
              sis     = mean(sis))
```

```
## # A tibble: 2 x 11
##     new   age agegp.1 agegp.2 agegp.3 agegp.4 female  male nwhite white   sis
##   <int> <dbl>   <dbl>   <dbl>   <dbl>   <dbl>  <dbl> <dbl>  <dbl> <dbl> <dbl>
## 1     0  39.5   0.105   0.426   0.341   0.127  0.143 0.857  0.468 0.532 0.475
## 2     1  39.4   0.109   0.416   0.356   0.119  0.174 0.826  0.473 0.527 0.437
```

One option is to reweight each arm of the trial to the target population based on covariates observed in both the trial and the target population. Since not all covariates we have used in the outcome model in the trial data analysis are observed in the target population, however, there is the risk that separate weighting of each trial arm may further distort between arms balance of such variables.

Therefore we use a two step weighting procedure: first reweighting the trial sample to obtain better balance between the two treatment arms, and second to weight this reweighted trial sample to the target population.

### 7.1.1 Reweight the trial sample to obtain better balance between treatment arms

```
new.mod <- glm(new ~ splines::ns(age, 9)*fem*nwhite*sis, family = binomial,
               data = sdat)
sdat <- sdat %>% bind_cols(new.ps = predict(new.mod, type = "response")) %>%
    mutate(new.wt = ifelse(new == 1, 1/new.ps, 1/(1-new.ps)),
           new.wt = new.wt / mean(new.wt)) %>%
    select(-new.ps)

sdat %>%
    group_by(new) %>%
    summarize(age     = weighted.mean(age, new.wt),
              agegp.1 = weighted.mean(1 * (agegp == 1), new.wt),
              agegp.2 = weighted.mean(1 * (agegp == 2), new.wt),
              agegp.3 = weighted.mean(1 * (agegp == 3), new.wt),
              agegp.4 = weighted.mean(1 * (agegp == 4), new.wt),
              female  = weighted.mean(fem, new.wt),
              male    = 1 - female,
              nwhite  = weighted.mean(nwhite, new.wt),
              white   = 1 - nwhite,
              sis     = weighted.mean(sis, new.wt),
              tot.wt  = sum(new.wt))
```

```
## # A tibble: 2 x 12
##     new   age agegp.1 agegp.2 agegp.3 agegp.4 female  male nwhite white   sis tot.wt
##   <int> <dbl>   <dbl>   <dbl>   <dbl>   <dbl>  <dbl> <dbl>  <dbl> <dbl> <dbl>  <dbl>
## 1     0  39.5   0.101   0.431   0.343   0.125  0.143 0.857  0.472 0.528 0.455   465.
## 2     1  39.4   0.108   0.420   0.354   0.118  0.146 0.854  0.478 0.522 0.458   468.
```

The between arms balance looks good. Let’s look at these weights.

```
sdat %>% 
    mutate(new = factor(new, labels = c("old", "new"))) %>%
    ggplot(aes(x = new.wt, group = new, color = new)) +
    geom_density(alpha = .1) +
    theme_bw()
```

They are not extreme, which is a good thing.

Note that this reweighted trial sample with better balance is perfectly suited to estimate treatment effects in the trial. We’ll fit the SATE model and the effect modification model to this dataset below. Note that we will pay attention to the point estimate, not the estimated variance; because the weights argument in package lme4 was not designed for probability weights, the point estimates are correct, but the estimated variances and degrees of freedom are not. Where we need those estimates, we will run the model in Stata, which deals with probability weights appropriately.

```
slong <- sdat %>%
    gather(post, cd4, -c(id, age, agegp, fem, nwhite, sis, new, new.wt)) %>%
    mutate(post = 1 * (post == "cd4.post"))

m.sate.adjusted <- 
    lmer(cd4 ~ age + fem + interaction(nwhite, sis) +
             new + age*new + fem*new + new*interaction(nwhite, sis) +
             post + age*post + fem*post + post*interaction(nwhite, sis) +
             new*post + (1|id),
         data = slong, weights = new.wt)
summary(m.sate.adjusted)$coef["new:post",]
```

```
##     Estimate   Std. Error           df      t value     Pr(>|t|) 
## 3.386079e+01 4.244422e+00 9.387142e+02 7.977715e+00 4.323802e-15
```

This SATE estimate is slightly smaller than that before reweighting.

```
m.effmod.adjusted <- 
    lmer(cd4 ~ age + fem + interaction(nwhite, sis) +
             new + age*new + fem*new + new*interaction(nwhite, sis) +
             post + age*post + fem*post + post*interaction(nwhite, sis) +
             new*post + new*post*interaction(nwhite, sis) + (1|id),
         data = slong, weights = new.wt)
summary(m.effmod.adjusted)$coef[19:22,]
```

```
##                                      Estimate Std. Error       df  t value   Pr(>|t|)
## new:post                             19.00278   7.688133 935.5915 2.471703 0.01362415
## interaction(nwhite, sis)1.0:new:post 21.41792  11.582468 935.5915 1.849167 0.06474901
## interaction(nwhite, sis)0.1:new:post 22.35354  11.853326 935.5915 1.885846 0.05962535
## interaction(nwhite, sis)1.1:new:post 20.33846  11.634496 935.5915 1.748117 0.08077186
```

The effect modification part of this model is very similar to that before reweighting. After all, the balance of the trial data was not perfect but was not bad before this reweighting adjustment.

### 7.1.2 Weight the trial sample to the target population.

```
stack <- bind_rows(
    sdat %>% 
        select(id, age, agegp, fem, nwhite, sis, new, 
               cd4.base, cd4.post, new.wt) %>%
        mutate(sample = 1),
    pop %>% 
        mutate(id = NA,
               age = NA,
               sis = NA,
               new = NA,
               cd4.base = NA,
               cd4.post = NA,
               new.wt = 1) %>%
        select(id, age, agegp, fem, nwhite, sis, new, 
               cd4.base, cd4.post, new.wt) %>%
        mutate(sample = 0)
)

sample.mod <- 
    svyglm(sample ~ factor(agegp)*fem*nwhite, family = quasibinomial,
           design = svydesign(ids=~1, weights = ~ new.wt, data = stack))

stack <- stack %>% 
    bind_cols(ps = predict(sample.mod, type = "response")) %>%
    mutate(sample.wt = ifelse(sample == 0, 1, (1-ps)/ps),
           wt = new.wt * sample.wt) %>%
    select(-ps) %>%
    group_by(sample) %>%
    mutate(wt = wt / mean(wt))

stack %>%
    group_by(sample, new) %>%
    summarize(age     = weighted.mean(age, wt),
              agegp.1 = weighted.mean(1 * (agegp == 1), wt),
              agegp.2 = weighted.mean(1 * (agegp == 2), wt),
              agegp.3 = weighted.mean(1 * (agegp == 3), wt),
              agegp.4 = weighted.mean(1 * (agegp == 4), wt),
              female  = weighted.mean(fem, wt),
              male    = 1 - female,
              nwhite  = weighted.mean(nwhite, wt),
              white   = 1 - nwhite,
              sis     = weighted.mean(sis, wt),
              tot.wt  = sum(wt))
```

```
## # A tibble: 3 x 13
## # Groups:   sample [?]
##   sample   new   age agegp.1 agegp.2 agegp.3 agegp.4 female  male nwhite white    sis tot.wt
##    <dbl> <int> <dbl>   <dbl>   <dbl>   <dbl>   <dbl>  <dbl> <dbl>  <dbl> <dbl>  <dbl>  <dbl>
## 1      0    NA  NA     0.341   0.309   0.247  0.103   0.266 0.734  0.639 0.361 NA     54220 
## 2      1     0  36.1   0.333   0.313   0.246  0.107   0.260 0.740  0.636 0.364  0.522   463.
## 3      1     1  35.8   0.349   0.304   0.247  0.0995  0.272 0.728  0.642 0.358  0.515   470.
```

This looks good! Let’s look at the weights.

```
stack %>%
    filter(sample == 1) %>%
    mutate(new = factor(new, labels = c("old", "new"))) %>%
    ggplot(aes(x = wt, group = new, color = new)) +
    geom_density(alpha = .1) +
    theme_bw()
```

Now we grab the weighted trial data

```
sdat <- stack %>% filter(sample == 1)

slong <- sdat %>%
    gather(post, cd4, 
           -c(id, age, agegp, fem, nwhite, sis, new, sample, 
              new.wt, sample.wt, wt)) %>%
    mutate(post = 1 * (post == "cd4.post"))
```

## 7.2 Weighted effect modification model for TATE estimation

Now we refit the effect modification model to this trial dataset that has been weighted to the target population. Again we are ignoring estimated variace here; we will get the correct estimates using Stata later.

```
m.effmod.wtd <- 
    lmer(cd4 ~ age + fem + interaction(nwhite, sis) +
             new + age*new + fem*new + new*interaction(nwhite, sis) +
             post + age*post + fem*post + post*interaction(nwhite, sis) +
             new*post + new*post*interaction(nwhite, sis) + (1|id),
         data = slong, weights = wt)

summary(m.effmod.wtd)$coef[19:22,]
```

```
##                                       Estimate Std. Error     df   t value     Pr(>|t|)
## new:post                             32.903852   8.918195 994.89 3.6895191 0.0002368559
## interaction(nwhite, sis)1.0:new:post  9.335134  11.745935 994.89 0.7947544 0.4269459063
## interaction(nwhite, sis)0.1:new:post  4.593148  13.509615 994.89 0.3399910 0.7339350510
## interaction(nwhite, sis)1.1:new:post  3.294465  11.154916 994.89 0.2953375 0.7677976585
```

Note that after the weighting to population, the fitted effect modification model has changed a lot. The coefficients of the interaction terms above are much smaller than before, and smaller than their under-estimated standard errors.

This is an indicator that the model was misspecified. This is not a cause for concern, because we can almost always be sure that the model is misspecified. Also, from the beginning, we sort of knew that modeling CD4 count change either on an additive or on a multiplicative scale is not right.

Anyway, this fitted model suggests we might be able to estimate TATE using a non-interaction moel

```
m.xzate <- 
    lmer(cd4 ~ age + fem + interaction(nwhite, sis) +
             new + age*new + fem*new + new*interaction(nwhite, sis) +
             post + age*post + fem*post + post*interaction(nwhite, sis) +
             new*post + (1|id),
         data = slong, weights = wt)

summary(m.xzate)$coef["new:post",]
```

```
##     Estimate   Std. Error           df      t value     Pr(>|t|) 
## 3.740766e+01 4.021817e+00 9.974117e+02 9.301185e+00 8.532915e-20
```

But we could also argue that a decision to abandon an effect modification model at this point is ad hoc, at least because the weighted model does not have power to reject effect modification. To use the weighted effect modification model is appropriate still because the TATE range it estimates carries with it all the uncertainty that results from increased variance due to weighting.

```
TATE.wtd <- contest(m.effmod.wtd, cbind(rbind(c(rep(0, 18), 1),
                                              c(rep(0, 18), 1)),
                                        pop.mean.nwhite * (1 - pop.mean.sis),
                                        (1 - pop.mean.nwhite) * pop.mean.sis,
                                        pop.mean.nwhite * pop.mean.sis),
                    joint = FALSE, check_estimability = TRUE)

TATE.wtd <- cbind(pop.mean.sis = pop.mean.sis, TATE.wtd)
TATE.wtd
```

```
##   pop.mean.sis Estimate Std. Error     df  t value    lower    upper     Pr(>|t|)
## 1          0.2 38.42776   4.813294 994.89 7.983671 28.98238 47.87313 3.900362e-15
## 2          0.6 37.54753   4.111727 994.89 9.131814 29.47887 45.61618 3.670858e-19
```

## 7.3 Correction of interval estimates using Stata

OK, there is a problem here. The number of degrees of freedom reported from the weighted model is larger than the sample size (933). The problem is that the weights argument in the lme4 package is not for probability weights. We wil fit the same model in Stata.

First, we write the data out as a Stata data file.

```
foreign::write.dta(slong, file = here::here("data","synthetictrial.dta"), version = 10)
```

Then in Stata, we run the code StataFits.do (to be placed in the same folder with file synthetictrial.dta).

Now to input by hand results from Stata.

```
sate.naive <- c(estimate = 36.55986,
                lower = 27.96777,
                upper = 45.15195)
    
    
sate.raw <- c(estimate = 35.80648,
              lower = 27.25114,
              upper = 44.36182)

tate.method1.raw <- cbind(pop.mean.sis = pop.mean.sis,
                          estimate = c(36.24252, 39.33251),
                          lower = c(25.85594, 30.03929),
                          upper = c(46.62911, 48.62573))

sate.adjusted <- c(estimate = 33.86079,
                   lower = 25.4003,
                   upper = 42.32128)

tate.method1.adjusted <- cbind(pop.mean.sis = pop.mean.sis,
                               estimate = c(34.16277, 37.11585),
                               lower = c(23.50098, 28.54771),
                               upper = c(44.82456, 45.68399))

xzate <- c(estimate = 37.40766,
           lower = 27.04435,
           upper = 47.77097)

tate.method2 <- cbind(pop.mean.sis = pop.mean.sis,
                      estimate = c(38.42776, 37.54753),
                      lower = c(25.98259, 27.38064),
                      upper = c(50.87292, 47.71441))
```

# 8 Plot results from both methods

```
## png 
##   2
```

```
par(mar = c(4.5, 5, 1, 1))
plot(NA, NA, xlim = c(-.1, .6), ylim = c(10, 69),
     cex.main = .9, cex.lab = .85, cex.axis = .8, xaxt = "n",
     ylab = "increase in CD4 count (cells/ml) \ndue to new (vs. old) treatment",
     xlab = "")
segments(-.03, sate.adjusted["lower"], -.03, sate.adjusted["upper"])
points(-.03, sate.adjusted["estimate"], pch=16)
segments(.07, xzate["lower"], .07, xzate["upper"], col = "gray40")
points(.07, xzate["estimate"], pch=16, col = "gray40")
axis(1,
     at     = seq(pop.mean.sis[1], pop.mean.sis[2], .1),
     labels = seq(pop.mean.sis[1], pop.mean.sis[2], .1)*100,
     tick = TRUE, cex.axis = .8)
axis(1,
     at = .4, tick = FALSE, cex.axis = .8, pos = 0,
     labels = "sensitivity parameter: percentage of target population\nwith severe immune suppression")
segments(pop.mean.sis[1], tate.method1.adjusted[1, "estimate"],
         pop.mean.sis[2], tate.method1.adjusted[2, "estimate"], col="blue", lwd=2)
segments(pop.mean.sis[1], tate.method1.adjusted[1, "lower"   ],
         pop.mean.sis[2], tate.method1.adjusted[2, "lower"   ], col="blue", lty=2)
segments(pop.mean.sis[1], tate.method1.adjusted[1, "upper"   ],
         pop.mean.sis[2], tate.method1.adjusted[2, "upper"   ], col="blue", lty=2)
segments(pop.mean.sis[1], tate.method2[1, "estimate"],
         pop.mean.sis[2], tate.method2[2, "estimate"], col="red", lwd=2)
segments(pop.mean.sis[1], tate.method2[1, "lower"   ],
         pop.mean.sis[2], tate.method2[2, "lower"   ], col="red", lty=2)
segments(pop.mean.sis[1], tate.method2[1, "upper"   ],
         pop.mean.sis[2], tate.method2[2, "upper"   ], col="red", lty=2)
text(c(-.03, .07, .02, .4), c(20, 21, 15, 60), adj = c(.5, .5, .5), cex = .8,
     labels = c("SATE", "(X,Z)-adjusted\nATE", "(95% confidence intervals)", "range of TATE (95% confidence bounds)\nblue: outcome-model-based method\nred: weighted-outcome-model-based method"))
```
